# Signaling pathways and therapeutic interventions in gastric cancer

**DOI:** 10.1038/s41392-022-01190-w

**Published:** 2022-10-08

**Authors:** Zi-Ning Lei, Qiu-Xu Teng, Qin Tian, Wei Chen, Yuhao Xie, Kaiming Wu, Qianlin Zeng, Leli Zeng, Yihang Pan, Zhe-Sheng Chen, Yulong He

**Affiliations:** 1 Guangdong Provincial Key Laboratory of Digestive Cancer Research, Digestive Diseases Center, Scientific Research Center, The Seventh Affiliated Hospital of Sun Yat-Sen University, 518107 Shenzhen, Guangdong China; 2 Department of Pharmaceutical Sciences, College of Pharmacy and Health Sciences, St. John’s University, Queens, NY 11439 USA; 3 Institute for Biotechnology, St. John’s University, Queens, NY 11439 USA

**Keywords:** Gastrointestinal cancer, Tumour biomarkers, Cancer therapy, Gastrointestinal cancer

## Abstract

Gastric cancer (GC) ranks fifth in global cancer diagnosis and fourth in cancer-related death. Despite tremendous progress in diagnosis and therapeutic strategies and significant improvements in patient survival, the low malignancy stage is relatively asymptomatic and many GC cases are diagnosed at advanced stages, which leads to unsatisfactory prognosis and high recurrence rates. With the recent advances in genome analysis, biomarkers have been identified that have clinical importance for GC diagnosis, treatment, and prognosis. Modern molecular classifications have uncovered the vital roles that signaling pathways, including EGFR/HER2, p53, PI3K, immune checkpoint pathways, and cell adhesion signaling molecules, play in GC tumorigenesis, progression, metastasis, and therapeutic responsiveness. These biomarkers and molecular classifications open the way for more precise diagnoses and treatments for GC patients. Nevertheless, the relative significance, temporal activation, interaction with GC risk factors, and crosstalk between these signaling pathways in GC are not well understood. Here, we review the regulatory roles of signaling pathways in GC potential biomarkers, and therapeutic targets with an emphasis on recent discoveries. Current therapies, including signaling-based and immunotherapies exploited in the past decade, and the development of treatment for GC, particularly the challenges in developing precision medications, are discussed. These advances provide a direction for the integration of clinical, molecular, and genomic profiles to improve GC diagnosis and treatments.

## Introduction

Gastric cancer (GC) remains one of the most common cancer types worldwide. According to the GLOBOCAN 2020 report, the global morbidity and mortality of GC rank fifth and fourth, respectively, with more than one million newly diagnosed cases and approximately one fatal case in every 13 cancer-related deaths.^1^ More than 95% of GC cases are adenocarcinomas.^2^ Men are twice as likely as women to suffer and die from GC.^3^ Despite a decline in the global prevalence and death rate of GC, rates remain high in Eastern Asian countries, which account for more than 70% of newly diagnosed and death cases of GC in the world.^1,4^ Notably, in both low-risk and high-risk regions, the incidence of GC is elevated in populations younger than 50 years, which may be linked to increased obesity and gastric microbiome dysbiosis associated with modern lifestyle.^5^ Thus, many challenges remain in controlling GC.

GC is generally categorized as cardia and non-cardia subtypes, which arise from the upper stomach and the mid-distal stomach, respectively. Each subtype has distinct epidemiological characteristics and risk factors.^6^ Non-cardia GC is more prevalent in Eastern Asian populations, while cardia GC is more common in Western countries.^7^ Chronic infection by *Helicobacter pylori* (*H. pylori*) is the dominant risk factor for the development of non-cardia GC.^8^ *H. pylori* infection, however, is generally not associated with cardia GC and may even reduce the risk of cardia GC in some populations.^9^ The molecular mechanism of *H. pylori* infection-mediated GC has not been completely elucidated. Prolonged *H. pylori* infection is thought to lead to chronic gastritis, where gastric acid secretion is inhibited by inflammatory mediators such as tumor necrosis factor-α (TNF-α) and interleukins. The loss of gastric acidity further exacerbates *H. pylori* infection and inflammation, causing parietal damage, ulcers, and atrophy of the stomach.^10,11^ Other contributors to non-cardia GC development include smoking tobacco, drinking alcohol, and consuming salt-preserved food or red/processed meat, which can cause destruction of stomach mucosa and enhance persistency of *H. pylori* infection.^12–14^ These factors are also associated with cardia GC,^15^ whereas obesity and gastroesophageal reflux disease are recognized as risk factors specifically linked to cardia but not non-cardia GC.^16^ In addition, infection with Epstein–Barr virus (EBV) is an important etiological agent responsible for ~10% of GC, frequently in male patients and the cardia subtype.^17^ EBV infection can promote the hypermethylation of tumor suppressor genes, inflammation of gastric mucosa, and immune evasion of the host, resulting in gastric carcinogenesis.^18^ As sustained infection with *H. pylori* and EBV can cause chronic inflammatory stress in the stomach, there is emerging attention to GC risk and co-infection by both pathogens, since *H. pylori* co-infection with EBV increases the occurrence of GC^19,20^ and may stimulate aggressiveness of GC.^21^

In addition to environmental and lifestyle factors, genetic aberrations (including gene mutations, chromosomal alterations, transcriptional dysregulations, and epigenetic modifications) are indispensable co-contributors in GC carcinogenesis.^22^ Approximately 10% of GC cases have a familial aggregation profile, and 1–3% have a confirmed hereditary mutation.^23^ The major type of hereditary GC is the autosomal dominant hereditary diffuse gastric cancer (HDGC) characterized by diffuse histopathological features. HDGC is frequently associated with a loss-of-function mutation in the *Cadherin-1* (*CDH1*) gene encoding E-cadherin, which is essential for cell–cell adhesion and maintenance of the epithelial cell phenotype. E-cadherin also plays vital roles in signaling pathways that regulate cell survival, proliferation, migration, and invasion.^24,25^ The link between the *CDH1* gene mutation and the diffuse type of GC was first identified in a large Aboriginal family in New Zealand in 1998 by Guilford and colleagues.^26^ Molecular genetic testing for the *CDH1* gene mutation is a recommended approach for confirming the diagnosis and family studies of HDGC.^27^

The treatment and prognosis for GC largely depend on cancer staging, which is usually evaluated using the American Joint Committee on Cancer (AJCC) tumor-node-metastasis (TNM) system. This system describes the extent of tumor invasion into the gastric wall layers (T category), the spread of the tumor to nearby lymph nodes (N category), and the migration of cancer cells to other organs (M category).^28^ The overall staging of GC is assigned from large staging groups after the combination of the TNM information, ranging from earliest stage 0 (carcinoma in situ) to stages I through IV; the larger number, the more advanced the cancer is with the larger extent of spread.^29^ Surgery is the primary approach for treating GC in all stages, especially for those in the early stage.^30^ Chemotherapy or chemoradiation is the main therapeutic intervention applied either before surgery to shrink the tumor or after surgery to kill any remaining cancer cells.^31^ For advanced GC patients with unresectable local cancer, recurrence, or metastasis, chemotherapy is usually the first-line treatment to control cancer progression for as long as possible, and a combination of chemotherapy with targeted therapy, immunotherapy, or radiation therapy may be adopted.^2^

Because GC is morphologically heterogeneous, decisions about therapy and predictions for patient survival rely on histopathological classifications. The traditional Lauren classification has been widely used in clinical practices since it was introduced in 1965. This classification divides GC into intestinal type with glandular growth pattern, diffuse type with poorly cohesive cells, and mixed type.^32^ The intestinal-type GC occurs more commonly in men and the elderly and is associated with *H. pylori*-related chronic gastritis as well as gastroesophageal reflux disease. The diffuse-type GC, usually with poorer clinical outcomes, is more prevalent in women and the younger populations and is more relevant to dysfunction in cell adhesion, as found in *CDH1*-mutated hereditary cases.^33^ The other broadly used histology classification is the World Health Organization (WHO) guidelines issued in 2010 and updated most recently in 2019, which characterizes GC as papillary, tubular, mucinous, and poorly cohesive types followed by several subdivisions under each category.^34^ Japanese pathologists also use the Nakamura classification or the Japanese Gastric Cancer Association (JGCA) classification, which can distinguish differentiated tumors from undifferentiated tumors.^35,36^ Although the histopathological classifications provide recommendations for surgery and chemotherapy selections, they are insufficient to guide personalized treatments for GC patients.

With the recent advances in genome analysis, biomarkers have been identified with clinical importance for GC diagnosis, treatment, and prognosis. These include molecules in growth factor pathways (e.g., the human epidermal growth factor receptor 2 (HER2)), regulators of the cell cycle and apoptosis (e.g., the tumor protein p53 (encoded by *TP53* gene)), cell adhesion factors (such as E-cadherin), immune checkpoint control modulators programmed death 1 and programmed death-ligand 1 (PD-1/PD-L1), and other molecules relevant to DNA, RNA, exosome, or epigenetic modifications.^37,38^ HER2 is the first clinically used molecular biomarker for GC patients. Approximately one-fifth of GC cases are HER2-positive, and determination of HER2 expression using immunohistochemistry (IHC) and fluorescence in situ hybridization (FISH) is mandatory for patients diagnosed with advanced GC.^39^ In 2010, the international Trastuzumab for Gastric Cancer (ToGA) phase III clinical study showed that the HER2 monoclonal antibody trastuzumab co-administered with cisplatin plus capecitabine or fluorouracil (5-FU) had better therapeutic outcomes compared to chemotherapy alone.^40^ Later in the same year, trastuzumab was approved by the United States Food and Drug Administration (FDA) as the first targeted drug used in combination with chemotherapeutic drugs for first-line treatment of HER2-positive metastatic GC.

To facilitate further development of personalized therapies for GC, molecular classifications have been introduced. Two large-scale, comprehensive genome-wide and molecular analyses on gastric tumors resulted in two major molecular classifications that partially overlap and complement. One proposed by The Cancer Genome Atlas (TCGA) research network in 2014 classified GC into four subtypes: EBV-positive (EBV^+^), microsatellite instable (MSI), genomically stable (GS), and chromosomal unstable (CIN).^41^ The Asian Cancer Research Group (ACRG) in 2015 classified GC into MSI, microsatellite stable or epithelial-mesenchymal transition (MSS/EMT), MSS positive for TP53 (MSS/TP53^+^), and MSS with loss of TP53 (MSS/TP53^−^) subtypes.^42^ Comprehensive molecular characterization of these GC subtypes shows clinical implications for GC treatment and prognosis (Table 1).^43,44^ With the development of immunotherapy in cancer management, the molecular classifications of GC have helped predict patients’ responsiveness to immunotherapy. Subgroups of GC patients with EBV^+^, high degree of MSI, or high burden of mutation are more likely to have a survival benefit from anti-PD-1 drugs like nivolumab and pembrolizumab.^43^

**Table 1:** Molecular characterizations and clinical implications of gastric cancer subtypes by TCGA and ACRG classifications

TCGA classification				
Subtypes	MSI (21.7%)	GS (19.7%)	EBV^+^ (8.8%)	CIN (49.8%)
Molecular characterizations	• High mutation rates and hypermethylation • Gene mutations of kinases: *EGFR*, *HER2/3*, *JAK2*, *FGFR2*, *MET*, *PIK3CA* • Expression loss of HLA class I complex and reduced antigen presentation to the immune system	• Alterations in cell adhesion-related genes: CDH1, RHOA, CLDN18-ARHGAP26 fusion • Upregulated angiogenesis-related pathways	• Frequent DNA hypermethylation • *CDKN2A* silencing • Mutations in *PIK3CA*, *ARID1A*, *BCOR*, *TP53* genes • Amplification of *JAK2* and *PD-L1/2* • Immune cell signaling enrichment	• Frequent *TP53* mutation • Gene amplification of receptor tyrosine kinases: *EGFR*, *HER2/3*, *JAK2*, *FGFR2*, *MET*, *PIK3CA*, *NRAS/KRAS*
Clinical implications	• Intermediate prognosis • Less sensitive to adjuvant chemotherapy • Sensitive to checkpoint inhibitor immunotherapy	• Poor prognosis • Less sensitive to adjuvant chemotherapy	• Good prognosis • Sensitive to checkpoint inhibitor immunotherapy	• Intermediate prognosis • Sensitive to adjuvant chemotherapy

ACRG classification				
subtypes	MSI (23%)	MSS/EMT (15%)	MSS/TP53^+^ (26%)	MSS/TP53^-^ (36%)
Molecular characterizations	• High mutation rates in *KRAS*, *ALK*, *ARID1A*, PI3K pathway • Frequent DNA hypermethylation • Loss of MLH1	• Low mutation rates • Loss of *CDH1*	• EBV positivity • Frequent mutations in *APC*, *ARID1A*, *KRAS*, *PIK3CA*, *SMAD4*	• Mutation or loss of TP53 • Gene amplifications of tyrosine kinase receptors like *HER2*, *EGFR*, and cell cycle regulators like *CCNE1*, *CCND1*, *MDM2*
Clinical implications	• Mostly diagnosed at early stages (I/II) • Good prognosis and lower frequency of recurrence	• Prevalent in the younger population • Diagnosis at advanced stages (III/IV) • Poor prognosis and high frequency of recurrence	• Intermediate prognosis and chance of recurrence	• Intermediate prognosis and chance of recurrence • High frequency of lymphovascular invasion

*TCGA* The Cancer Genome Atlas, *ACRG* Asian Cancer Research Group, *MSI* microsatellite instable, *EBV* Epstein–Barr virus, *GS* genomically stable, *CIN* chromosomal unstable, *MSS* microsatellite stable, *EMT* epithelial-mesenchymal transition, *TP53* tumor protein p53, *EGFR* epidermal growth factor receptor, *HER2/3* human epidermal growth factor receptor 2/3, *JAK2* Janus kinase 2, *FGFR2* fibroblast growth factor receptor 2, *MET* mesenchymal-epithelial transition factor, *PIK3CA* phosphatidylinositol-4,5-bisphosphate 3-kinase catalytic subunit alpha, *HLA* human leukocyte antigen, *CDKN2A* cyclin-dependent kinase inhibitor 2A, *ARID1A* AT-rich interactive domain-containing protein 1A, *BCOR* B-cell lymphoma 6 corepressor, *PD-L1/2* programmed death-ligand 1/2, *CDH1* - cadherin 1, *RHOA* Ras homolog family member A, *CLDN18* Claudin 18, *ARHGAP26* Rho GTPase Activating Protein 26, *NRAS* neuroblastoma RAS viral oncogene homolog, *KRAS* Kirsten rat sarcoma viral oncogene homolog, *ALK* anaplastic lymphoma kinase, *PI3K* phosphoinositide 3-kinase, *MLH1* MutL Homolog 1, *APC* adenomatous polyposis coli, *SMAD4* mothers against decapentaplegic homolog 4, *CCNE1 & CCND1* cyclin E1 & D1, *MDM2* Mouse double minute 2 homolog

The identification of biomarkers and molecular classification have also provided important clues to improve early diagnosis and therapeutic interventions for rare GC types with unique histopathological characteristics, such as gastric signet-ring cell carcinoma (GSRCC). GSRCC is classified into diffuse, undifferentiated, and poorly cohesive types, noted for their poorly cohesive single cells and absence of gland formation.^45^ There are many clinical challenges in the diagnosis and treatment of GSRCC. GSRCC exhibits distinct epidemiology, oncogenesis processes, and therapeutic sensitivity profiles compared to other subtypes of diffuse GC.^46,47^ Moreover, GSRCC cases are frequently diagnosed at an advanced stage, in part because of the impracticality of using endoscopy and the lack of pathological tests for early stage screening.^48^ The regimen for treating GSRCC is still controversial, and overtreatment with chemotherapy may occur with detrimental results because of this lack of adequate predictive biomarkers.^49^ Since mutations in the *CDH1* gene^50^ and high *CLDN18-ARHGAP 26/6* fusion^51^ have been reported in GSRCC patients, GSRCC is considered a GS subtype of TCGA molecular classification,^49^ and the high CLDN18.2 expression found among advanced GSRCC patients has provided a novel therapeutic option of CLDN18.2-targeted therapy.^52^ In addition, high MSI was found in 3.5% of GSRCC, and this specific group of GSRCC patients may benefit from immunotherapy using PD-1 inhibitors.^53,54^

**Fig. 1:**
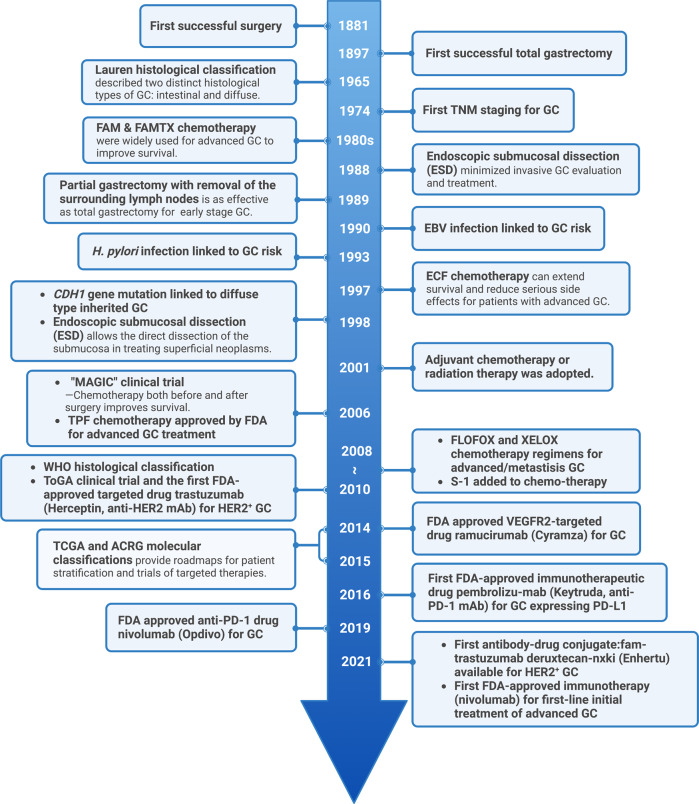
Timeline of selected key findings and significant therapy developments in gastric cancer. The major milestones for risk factor identification, classification and staging, and therapy developments for GC are listed. Chemotherapy regimens: FAM: fluorouracil (5-FU) + mitomycin C + doxorubicin; FAMTX: methotrexate + 5-FU + doxorubicin; ECF: epirubicin + cisplatin + 5-FU; TPF: docetaxel + cisplatin + 5-FU; FLOFOX: oxaliplatin + 5-FU + leucovorin; XELOX: capecitabine (Xeloda) + oxaliplatin; S-1: tegafur (5-FU prodrug) + 5-chloro-2,4-dihydroxypyridine (CDHP) + oteracil potassium (Oxo), in a molar ratio of 1:0.4:1. EBV Epstein–Barr virus, TCGA The Cancer Genome Atlas, ACRG Asian Cancer Research Group. This figure was created with Biorender.com

Since the first successful gastric resection in the 1880s, there has been tremendous progress in diagnosis and therapeutic strategies (Fig. 1) and significant improvements in patient survival in the long combat against GC. However, because GC is often asymptomatic until it progresses to higher malignancy levels, cases are often diagnosed at advanced stages, leading to unsatisfactory prognosis and high recurrence rates. The 5-year survival rates are as high as 68–80% for stage I GC, and then decrease sharply as the diagnosed staging becomes advanced, to 46–60% for stage II, 8–30% for stage III, and only 5% for stage IV.^55^ Resistance to chemotherapy and targeted drugs contributes to poor survival in GC.^56,57^ Therefore, identifying new biomarkers for early diagnosis and therapeutic selectivity and sensitivity is the main challenge in GC management. The modern molecular classifications support the important roles of signaling pathways like EGFR/HER2, p53, PI3K, immune checkpoint pathways, and cell adhesion signaling molecules in GC tumorigenesis, progression, metastasis, and therapeutic responsiveness. Four targeted drugs and two immune checkpoint inhibitors have already been approved by the FDA for GC treatment. Still, the relative significance of these signaling pathways in GC, their temporal activation and interaction with GC risk factors, and crosstalk among them is not well understood. There has been increasing attention to signaling pathways and the identification of novel therapeutic targets in GC research. In this article, the regulatory roles of signaling pathways in GC and potential biomarkers or therapeutic targets are reviewed. Furthermore, the current GC treatment and the development of signaling pathway-based targeted or immunotherapies will be discussed.

## Signaling pathways in gastric cancer and therapeutic implications

### MAPK signaling pathway

The mitogen-activated protein kinase (MAPK) signaling pathway is one of the most complicated cellular pathways involved in GC progression, including proliferation, migration, invasion, and metastasis.^58^ MAPKs are a large family of serine/threonine protein kinases that are responsible for cellular response to multiple extracellular stimuli.^59^ Each canonical single MAPK cascade pathway consists of at least three core kinases: MAPKKKs, MAPKKs, and MAPKs.^60^ The MAPK signaling pathway is shared by five cascades, which are accordingly named after the components of each MAPK tier: the extracellular signal-related kinases ERK (ERK1/2), Jun amino-terminal kinases (SAPK/JNK1,2,3), p38-MAPK (p38α, p38β, p38γ, and p38δ), ERK5, and ERK3/4.^61^

The MAPK/ERK signaling cascade is triggered by binding of extracellular factors to receptors including tyrosine kinases (RTKs), EGFR, and G protein-coupled receptors (GPCRs), and is sometimes triggered by vascular endothelial growth factor and its receptor (VEGF/VEGFR). Under physiological conditions, MAPK signaling is triggered through the activation of RAS proteins (KRAS, HRAS, and NRAS), a family of small guanine triphosphatases (GTPases) that integrate signals from a collection of upstream factors.^62^ RTK-RAS signaling pathway alterations are reported in about 37% of GC.^63^ In its GTP-bound activated condition, RAS undergoes a conformational shift in the switch I and II regions, which facilitates interactions with a variety of downstream effectors, including the RAF family of kinases (ARAF, BRAF, and CRAF).^64,65^ *BRAF* mutation occurs in all types of cancers and up to 11% in GC.^66^ Once activated, RAF kinases phosphorylate and activate MEK1/2 kinases, which in turn activate ERK1/2 kinases.^67^ ERK1/2 are vital sensors of proliferation, differentiation, and survival signals.^68^ Elevated p-ERK1/2 is an independent prognostic factor of poor survival in GC cases.^69^ The activated ERK1/2 kinases then phosphorylate a series of substrates that conduct critical biological processes.^68,70^ In GC, the MAPK/ERK pathways are involved in the regulation of cell motility by coordinating the activity of MMPs, cell adhesion, and EGFR-induced disassembly of focal adhesions, thus governing cell migration and invasion.^59,71^ Generally, the ERK3/4 MAPKs are considered atypical because of the absence of a tyrosine residue and the presence of the Ser-Glu-Gly motif in their activation loop.^72^ ERK5 can be activated by growth factors and oxidative stress and is essential for cell survival, normal development of the early embryo, and the vascular system.^73^

The JNK subgroup of MAPKs is encoded by three distinct genes: *MAPK8* (which encodes JNK1), *MAPK9* (which encodes JNK2), and *MAPK10* (which encodes JNK3).^74^ The JNK1/2 subtypes are ubiquitously expressed, whereas JNK3 is expressed primarily in the heart, brain, and testis.^75,76^ JNKs are activated by stress signals and proinflammatory stimuli such as heat shock and oxidative stress. MKK4 and MKK7 kinases are the upstream regulators of JNKs. Activated JNKs subsequently phosphorylate downstream c-Jun and JunD and activate transcription factors.^77^ An important JNK target is the transcription factor activating protein-1 (AP-1).^78^ Activation of JNKs leads to cell proliferation, apoptosis, or transformation.^79^ Interactions can occur between JNKs and the other MAPK pathways; JNK subtypes can activate p38-MAPK, while several upstream regulators in the p38-MAPK module are shared by the JNK isoforms. Studies have shown that JNK1/2 is involved in the sensitization of p38-MAPK inhibition to cisplatin-induced cell death, and the elevated level of reactive oxygen species (ROS) mediates the activation of JNK1/2 by P38-MAPK inhibition.^80^ Compared to wild-type controls, JNK1 knockout mice showed a significant decrease in gastric carcinogenesis mediated by N-methyl-N-nitrosourea.^81^ Consequently, JNK1 is involved in tumor initiation as well as progression and is a promising target for the prevention of GC.

**Fig. 2:**
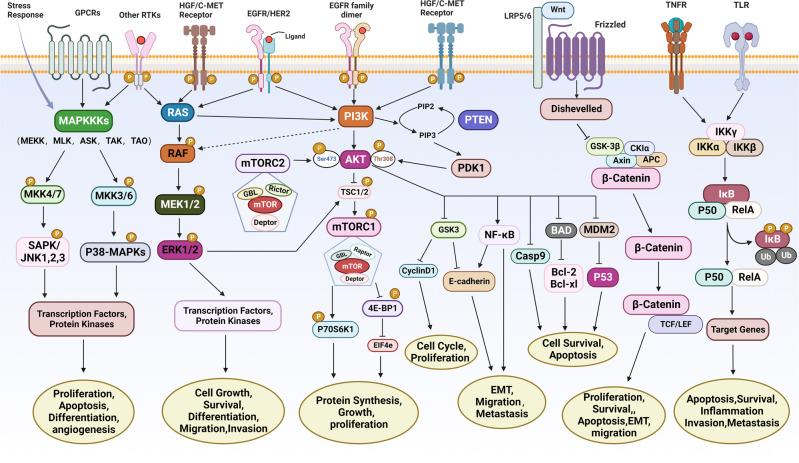
Main signaling pathways and fundamental factors in gastric cancer. The major signaling and crosstalk of MAPK, HER2, PI3K/AKT/mTOR, HGF/c-Met, p53, Wnt/β-catenin, and NF-κB pathways, as well as their regulatory roles in cellular processes, are illustrated. GPCRs G-protein-coupled receptors, HGF hepatocyte growth factor, c-MET c-mesenchymal-epithelial transition factor, EGFR epidermal growth factor receptor, HER2/3/4 human epidermal growth factor receptor 2/3/4, MAPKKKs mitogen-activated protein kinase kinase kinases, RTKs receptor tyrosine kinases, RAS rat sarcoma, RAF rapidly accelerated fibrosarcoma, MKK mitogen-activated protein kinase kinase, SAPK/JNK jun amino-terminal kinase, p38-MAPKs p38 group of mitogen-activated protein kinases, MEK mitogen-activated protein kinase kinase, ERK1/2 extracellular signal-related kinase 1/2, PI3K phosphoinositide 3-kinase, AKT protein kinase B, mTORC1/2 mammalian target of rapamycin complex 1/2, PTEN phosphatase and tensin homolog, PDK1 phosphoinositide-dependent protein kinase 1, TSC1/2 tuberous sclerosis complex 1/2, p70S6K1 phosphorylation of ribosomal p70S6 kinase 1, 4E-BP1 eukaryotic translation initiation factor 4E (eIF4E)-binding protein 1, NF-κB nuclear factor kappa-B, GSK3 glycogen synthase kinase 3, BAD Bcl-xl/Bcl-2-asociated death promoter, Casp9 cysteinyl aspartate specific proteinase 9, MDM2 murine double minute 2, p53 tumor protein 53, EMT epithelial-mesenchymal transition, LRP5/6 low-density lipoprotein receptor-related protein 5/6, CKIα casein kinase Iα, APC adenomatous polyposis coli, TCF/LEF T-cell factor/lymphoid enhancer factor, TNFR tumor necrosis factor receptor, TLR toll-like receptors, IKK IκB kinase. This figure was created with Biorender.com

The p38-MAPK is selectively activated by upstream MAPK kinase (MKK) 3 and MKK6 kinases.^82^ The major downstream targets of p38-MAPK are protein kinases and transcription factors such as MAPK-activated protein kinase 2 (MK2), mitogen- and stress-activated protein kinase 1 (MSK1), p53, transcription factor ELK1, and activating transcription factor 2 (ATF2).^83^ The p38-MAPK pathway features a complicated regulation in cancers. Several studies showed that p38 acts as an oncogenic factor and plays a key role in pathological events related to tumor progression, such as inflammation, invasion, and angiogenesis^84,85^ (Fig. 2). Activation of the p38-MAPK/AP-1 pathway is positively related to chemotherapy resistance in human GC cells.^86^ On the other hand, a wealth of evidence supports the role of p38-MAPK as a tumor suppressor, inducing cell apoptosis by way of the activation of p53.^87,88^ Cell cycle arrest is another possible consequence of tumor suppression by p38, carried out by downregulating ERK and JNK signaling pathways, thus restricting RAS transformation.^89^

RAS/RAF/MAPK and PI3K/AKT/mTOR signal transduction pathways are the most dysfunctional pathways in multiple cancer types including GC.^90,91^ RTKs alterations in tumors lead to activation of both MAPK and PI3K pathways, and targeting the PI3K pathway was confirmed to promote cancer progression through MAPK signals and vice versa^92^ (Fig. 2). *RAS* mutations are the most common MAPK alterations observed in human cancer.^93^ The mutation frequency of *KRAS* in GC is 6.5%, and *PIK3CA* is 25%.^94,95^ Generally, the *KRAS* mutation is found in intestinal-type tumors whereas the *NRAS* mutation is reported to appear in diffuse and metastatic GC.^96^ Using pathway-based gene set enrichment analysis, MAPK/ERK gene features were found elevated in the intestinal subtype of GC. Genes involved in the RAS/ERK signaling cascade, including *KRAS*, *EGFR*, *HER2*, and *MET*, have been found amplified in a mutually exclusive manner in about two out of five GC patients.^97^

Migration and invasion of GC cells mediated by the MAPK/ERK signaling pathway involves various other factors.^98–100^ For example, Spondin 2 (SPON2) promotes the EMT of GC cells by activation of the MAPK/ERK1/2 pathway and consequently accelerates the metastasis of GC. Chemerin may act as a pro-invasive factor via induction of VEGF, IL-6, and matrix metalloproteinase-7 (MMP-7) in GC, and the process relies on the phosphorylation of ERK1/2.^101^ ERK also mediates GC migration and invasion by regulating the activity of downstream proteins like MMPs.^71^ Other studies have demonstrated that RAS/MAPK signal transduction is involved in the proliferation of GC cells.

Recent studies have shown that epigenetic regulation can affect GC cell growth and metastasis through MAPK/ERK pathways.^102^ Micro RNAs (miRNAs) are multipotent in the regulation of various cellular pathways and play a fundamental role in tumor biology. In particular, they have been found to regulate MAPKs like ERK1/2 and JNK and to modulate proliferation, survival, and metastasis of GC cells.^103^ miR-592 overexpression has been identified to promote proliferation, migration, and invasion of GC by targeting Sprouty 2 (SPRY-2) through the MAPK/ERK and PI3K/AKT signaling pathways.^104^ In addition to miRNAs, some long non-coding RNAs (lncRNAs) are involved in tumorigenesis and the progression of GC mediated by the MAPK/ERK signaling pathway.^105^ For example, lncRNA CASC2 suppresses the proliferation of GC cells by regulating the ERK1/2 and JNK/MAPK signaling pathways.^106^

### HER2 signaling pathway

The frequency of HER2-positive tumors ranges from 4.4% to 53.4% in gastric/gastroesophageal cancer,^107,108^ and HER2-positive tumors are generally associated with more aggressive cancer and tumor recurrence.^109,110^ HER2 amplification/overexpression has been confirmed to play a critical role in GC tumorigenesis and development,^111^ and is a therapeutic target and biomarker for GC patients.^112^ The *HER2* gene, also known as receptor tyrosine-protein kinase erbB-2, p185, or neu, is located on the human chromosome 17 (17q12),^113^ and is a member of the epidermal growth factor receptor (EGFR) family of receptor tyrosine kinases. The EGFR family consists of four members, HER1 (ERBB1, EGFR), HER2 (ERBB2), HER3 (ERBB3), and HER4 (ERBB4),^114^ all of which are identified to participate in regulating tumor cell growth, proliferation, and migration. Although the four human HER genes are located on different chromosomes, all of them are composed of an intracellular domain with tyrosine kinase properties, a lipophilic transmembrane domain, and a cysteine-rich extracellular domain containing the ligand-binding pocket.^115^

EGFR family members exist as monomers on the cell surface, but dimerize once the ligand binds to the extracellular domain, followed by the transphosphorylation of intracellular domains.^116^ The binding of ligands to the extracellular domain of HER1, HER3, and HER4 leads to the formation of kinase-active hetero-oligomers.^117^ Specific ligands for HER2 have not been identified, though it becomes constitutively activated following its heterodimerization with other family members (HER1 and/or HER3),^118^ thereby triggering different and complicated signal transduction cascades. Moreover, spontaneous formation of various heterodimers increases with amplification of the *HER2* gene.^119^ Heterodimers containing HER2 provide a stronger signal and have significantly higher ligand-binding affinity than homodimers or heterodimers with other family members. For instance, in several HER2-induced cancers, the HER2/HER3 dimer, the most potent EGFR family heterodimer, is indispensable for tumorigenesis and tumor maintenance.^120^ Therefore, restricting the dimerization of HER2 with other EGFR family members, particularly HER3, might provide an efficient treatment strategy for HER2-positive tumors.

HER1 and HER2 are overexpressed in a heterogeneous manner in GC. HER3 and HER4 have also been detected in 20.7% and 13.3% of GC, respectively.^121^ Several studies proved the negative correlation between high HER3 expression levels and survival of GC patients.^122^ HER2 overexpression was also found to be a poor prognostic indicator in GC.^109,123^ HER2 overexpression drives tumorigenesis through the formation of spontaneous receptor homodimers, or heterodimers with other EGFR family members, resulting in activated downstream signaling cascades, such as PI3K/AKT/mTOR and MAPK/ERK1/2.^124,125^ This promotes tumor cell proliferation, differentiation, survival, angiogenesis, and metastasis^125–127^ (Fig. 2). For example, the HER2/HER3 heterodimers transduce PI3K signaling through direct binding of HER3 and the p85 subunit of PI3K.^128^

Trastuzumab (Herceptin), the first anti-HER2 monoclonal antibody targeting the extracellular domain of the HER2 protein, has been an acknowledged treatment for both early stage and metastatic HER2-positive breast cancer for decades.^129^ Trastuzumab interferes with HER2 signaling in tumors via various mechanisms: inhibition of dimerization, antibody-dependent cellular cytotoxicity, receptor internalization and/or degradation, and suppression of the PI3K/AKT/mTOR signaling cascades. Trastuzumab was also the first targeted agent approved as standard treatment for HER2-positive advanced GC based on the results of the ToGA trial.^40^ In the ToGA trial, it was found that there existed primary and secondary resistance to HER2 blockage in GC patients. Several potential mechanisms may explain this: alteration in HER2 dimers; activation of downstream signaling pathways such as PI3K/AKT, mTOR, and MAPK/ERK; and absence of downstream regulators or alternative transduction pathway from the insulin-like growth factor receptor (IGFR).^130^ In 2017, Deguchi et al.^131^ investigated HER2 expression and the occurrence of phosphatase and tensin homolog (PTEN) loss or *PI3K* mutation in 264 GC cases and reported the absence of PTEN in 34.5% of HER2-positive patients. No response was observed in patients with PTEN deficiency who received trastuzumab. PTEN deficiency and/or PI3KCA mutation leads to abnormal activation of the downstream AKT/mTOR signaling cascade, leading to ineffective inhibition of HER2.^132^ A peptidomimetic that binds extracellular subdomain IV and a nucleic-acid aptamer that binds the extracellular domain of HER2 have been found to downregulate the HER2-dependent signaling pathways, providing a promising novel treatment of HER2-positive GC and other tumors.^133,134^ In brief, a comprehensive understanding of the complicated interplay between the EGFR family and downstream signaling pathway cascades would assist in identifying patients who might benefit from EGFR family targeted therapies.

### PI3K/AKT/mTOR signaling pathway

The phosphoinositide 3-kinase (PI3K) pathway plays a key role in the proliferation and survival of various cancer cells including GC.^135–137^ The PI3K/AKT/mTOR signaling pathway promotes tumor progression in GC through several mechanisms, including the inhibition of apoptosis, induction of drug resistance, metastasis, and angiogenesis^138^ (Fig. 2). PI3K/AKT/mTOR pathway alteration plays a vital part in resistance to HER2-targeted therapy and chemoresistance in GC and several other solid tumors.^127,139,140^

PI3K is a broad family of lipid kinases consisting of three different classes (I, II, and III) that stand at the top of the PI3K/AKT/mTOR cascade.^141^ Class I PI3K is categorized into class IA and IB and is more tightly related to tumor progression.^142^ Classes II and III PI3Ks have been identified to contribute to the regulation of mTOR activation and autophagy.^143^ The activation of PI3Ks is triggered by the binding of a variety of ligands to the oncogenic receptor tyrosine kinases including EGFR, IGFR, PDGFR (platelet-derived growth factors receptor), and other growth factors.^135,136,144^ Activated PI3K catalyzes the phosphorylation of phosphatidylinositol diphosphate (PIP2) to phosphatidylinositol 3-phosphate (PIP3), which subsequently interacts with homology domain-containing proteins on the inner surface of the plasma membrane, resulting in conformational changes of downstream proteins.

AKT, also known as protein kinase B (PKB), normally exists in the cytoplasm.^145^ Upon activation of PI3K and PIP2, downstream AKT kinase translocates to the cell membrane, resulting in its conformational activation.^146^ AKT contains a central kinase domain with a threonine residue responsible for binding to the phosphoinositide-dependent protein kinase 1 (PDK1) and a C-terminal tail domain responsible for binding to the mammalian target of rapamycin complex 2 (mTORC2).^147^ While phosphorylation by PDK1 at Thr308 is fundamental, the activation of AKT also relies on phosphorylation by mTORC2 on Ser473.^148,149^ Phosphorylated AKT (p-AKT) plays an important part in the regulation of intracellular biological processes such as cell growth, survival, proliferation, apoptosis, EMT, metastasis, and angiogenesis.^147^ The lipid phosphatase and tensin homolog (PTEN), a well-known tumor suppressor gene that encodes a lipid phosphatase, is a negative regulator of PI3K signal conduction by converting PIP3 back to PIP2.^150^ PTEN dysfunction leads to constitutive activation of PI3K/AKT and downstream signaling, thereby stimulating cell proliferation and survival.^151,152^

mTOR is a highly conserved serine/threonine kinase that participates as an effector in the PI3K/AKT pathway.^153^ mTOR consists of two distinct functional complexes known as mTORC1 (mTOR, Raptor, and mLST8) and mTORC2 (mTOR, Rictor, mLST8, and mSIN1).^154^ Activation of both mTOR complexes is a vital consequence of RTK-based signaling transduction in tumors.^155^ The mTORC1 complex controls protein synthesis and cell growth by triggering the phosphorylation of ribosomal p70S6 kinase 1 (S6K1) at Thr229 and Thr389 and inactivating 4E-BP1 through direct phosphorylation.^156,157^ Activated S6K1 acts as a negative regulator and downregulates the PI3K pathway, subsequently suppressing adapter molecule insulin receptor substrate 1 (IRS-1), which obstructs the signaling between insulin growth factor 1 (IGF1) and PI3K.^158^ The inactivation of 4E-BP1 leads to a release of EIF4e from the dimer that triggers transcription of multiple genes.^159^ Activated AKT can interrupt the stable heterodimer tuberous sclerosis complex (TSC1/TSC2) by phosphorylating TSC2, thereby promoting the activity of mTORC1.^158^ In the progression of cancer, the activity of the PI3K/AKT pathway is elevated, and TSC1/TSC2 heterodimer is restrained by activated AKT, leading to mTORC1 activation and subsequent activation of the downstream factors (P70S6K1 and EIF4e).^160,161^ Another important substrate of AKT is GSK3, which promotes cell proliferation by regulating the production of cell cycle proteins like cyclin D1.^162^ AKT deactivates GSK3 by phosphorylation as well. GSK3 collaborates with mTORC1 by phosphorylating p70S6K1 at Ser371, which enhances mTORC1-mediated p70S6K1 phosphorylation on Thr389.^163^ Rictor is a critical component of mTORC2 and can function as a downstream substrate of GSK3.^164^ Alteration of mTORC2/Rictor influences the structure of actin and promotes cell proliferation by phosphorylating the downstream molecules^165,166^ (Fig. 2).

The PI3K/AKT/mTOR pathway is frequently altered in GC.^108,167^ From the TCGA molecular subtypes, most of the GC cases studied had different degrees of mutations in the *PIK3CA* gene and amplification of *RTK* genes such as *EGFR* and *HER2*.^41,168,169^ Mutations of the *PIK3CA* gene are likely to be late and isolated events in GC.^95,170^ The relationship between *PIK3CA* mutation and the prognosis of GC patients is controversial. Some reports identified that *PIK3CA* mutation promotes the risk of tumor aggressiveness, and the mutation in the exon 9 of *PIK3CA* has been identified as a helpful indicator for predicting prognosis in EBV-positive GC.^171–173^ Other studies declared no effective association between PIK3CA mutations and clinical outcome.^174,175^

Genomic amplification plays an important part in neoplastic progression. Amplification in *PIK3CA* is tightly associated with tumor progression, prognosis, and the emergence of drug resistance in GC.^176^ The amplification of *PIK3CA* leads to the elevation of AKT and p-AKT, thereby promoting migration, invasion, and lymph node metastasis in GC.^176^ LY294002, one specific inhibitor of PI3K, has been found to inhibit the activity of the ATP binding site of PI3K and lead to the reduction of p-AKT, which was closely associated with the proliferation and apoptosis of GC cells.^177^ Recently, APY0202, a small-molecule inhibitor of PIKfyve, has been found to be involved in inducing repression of autophagy and cell cycle arrest in an in vitro GC cell model, GC organoid model, and in vivo xenograft GC model.^178^

AKT acts as a central character in the activation of the PI3K axis.^179,180^ Elevated AKT and p-AKT expression was observed in over 74% of GC.^181^ The abnormal expression of p-AKT was closely related to PI3K and HER2 overexpression, and the high p-AKT level was identified as a hallmark of tumor progression, metastasis, and poor prognosis in GC.^182,183^ Lymphangiogenesis plays a crucial role in metastasis, recurrence, and prognosis in early GC.^184^ A previous study confirmed that p-AKT plays a significant role in the angiogenesis of GC via VEGF-A activation.^185^ Subsequently, several studies proved that inhibition of p-AKT/p-mTOR in vitro leads to a remarkable decrease of VEGF-C and VEGF-D in gastric tumor cells, and the authors proposed that lymphangiogenesis of GC might be efficiently regulated by the AKT/mTOR/VEGF-C/VEGF-D signaling pathway.^186^ mTOR can be activated via multiple upstream factors and acts as a bridge in a variety of downstream signaling pathways. mTOR stands at the terminus of the PI3K/AKT/mTOR signaling cascade and is one of the most independent elements of the PI3K axis.^187^ The mutations in upstream regulators from the different axes, such as EGFR, PI3K, and PTEN, can lead to over-activation of mTOR.^188–190^ Aberrant activation of mTOR has been detected in over 60% of GC cases.^191^ The dysregulation of mTOR activity participates in the regulation of GC cell growth and differentiation.^167^ In addition, some previous studies have identified that the expression of mTOR was much higher in GC tissues than in normal gastric tissues.^192^ Additionally, a positive link between elevated mTOR levels and pathological parameters like invasive depth and lymph node metastasis was found in GC.^193^ Therefore, mTOR expression can serve as a biomarker of not only the diagnosis of GC but also the invasiveness and metastasis of the tumor, and its prognostic role has been proven by the negative correlation with five-year survival rates of GC patients in cohort studies.^193,194^

The significant contribution of the PI3K/AKT/mTOR signaling pathway in the progression of GC suggests that this signal axis is a promising target for cancer therapy. From the results of existing clinical investigations in GC, the efficacy of PI3K inhibitors, AKT inhibitors, mTOR inhibitors, and other monotherapy were not as effective as dual PI3K/mTOR inhibitors or several combination therapies,^195^ suggesting that the restriction on the therapeutic effect by the heterogeneity of GC should be emphasized in designing new targeted medication regimens.

### P53 signaling pathway

The main role of p53 lies in its involvement in the regulation of DNA repair as well as in the control of the cell cycle, apoptosis, and differentiation, which is mainly through DNA-protein and protein-protein interactions.^196^ It can induce aging or promote cell apoptosis and DNA repair,^197^ providing a mechanism to prevent the accumulation of potentially malignant or defective cells.^198^ In vertebrates, p53 can temporarily block the cell cycle by regulating checkpoints in G1/S and G2/M phases^199^ and these regulatory processes are closely related to the transcriptional activation of related genes by the p53 protein. Cyclins and cyclin-dependent kinases (CDKs) are the two major proteins involved in cell cycle progression.^200^ Functional analysis revealed that Reprimo (RPRM) is transcriptionally regulated by p53 and serves to arrest the cell cycle at the G2/M checkpoint, by inhibiting nuclear translocation of the Cdc2/cyclin B1 complex.^201^ Significant downregulation of RPRM has been described in GC cells expressing wild-type p53.^202^ With DNA damage, the cell cycle is arrested in the G2/M phase as monitored by p53-mediated downregulation of p21, which prevents the transmission of mutagenic damage.^200^

p53 is affected by many non-coding RNAs. For example, miR-181a can elevate the expression and activity of p53^203^ by targeting the tumor suppressor ataxia-telangiectasia mutated (*ATM*) gene.^204^ miR-650 enhances the function of p53 in gene transcription and promotes cell growth by the upregulating expression of the inhibitor growth family member 4 (ING4).^205^ TP53-inducible nuclear protein 1 (TP53INP1) is a key element in p53-mediated cell death and cell cycle arrest. The upregulation of both miR-17-5p and miR-20a in GC can promote cell growth by deregulating TP53INP1 and p21.^206^ In contrast, miR-499 can indirectly upregulate p53 and its downstream target p21, activating caspase-apoptosis pathways.^207^ Therefore, downregulation of miR-449 observed in GC cells is associated with cell survival advantages.^207^ Mutations in some key sites of the p53 gene can directly lead to abnormal cell proliferation, while polymorphisms at non-important functional regions of *TP53* may also affect GC tumorigenesis.^208^ Studies have reported elevated expression levels of p53 in more than 75% of GC patients, and the mutation rate of the *TP53* gene in all GC patients is ~30%, but it may vary in patients with different GC subtypes and etiologies.^209,210^ The polymorphism of codon 72 of the *TP53* gene is closely associated with gastric carcinogenesis in the US population.^211^ *TP53* gene mutation is the main reason for the loss of normal function of p53 protein,^210,212^ which is an important initiating factor for the occurrence and development of GC. Cell cycle regulators, especially p16^INK4A^ (cyclin-dependent kinase inhibitor 2A, CDKN2A), are upregulated by p53 inactivation in precancerous GC and act as a barrier to disease progression.^213^ Co-deletion of *CDKN2A* and *TP53* in dysplastic gastric organoids promotes the cancer phenotype and also induces replication stress, thereby exposing susceptibility to inhibitors of the DNA damage response.^213^ In humans, folic acid (vitamin B9) supplementation may play a vital role in the chemoprevention of GC since it can significantly increase the expression of p53 and decreases the expression of the Bcl-2 oncogene protein in the gastric mucosa.^214,215^

*H. pylori* infection can promote the accumulation of mutations in the *TP53* gene, which has been reported to occur in 50% of gastric tumors.^216^ The proteasomal degradation of p53 may also be induced indirectly by *H. pylori* infection.^217,218^ In response to genotoxic stress, p53 triggers signaling pathways that lead to temporary cell cycle arrest, activating the repair process of DNA.^219^ Inactivation of p53 promotes genomic instability, which is a hallmark of cancer.^220^ Thus, inhibition of p53 can be a strategy for modulating host cell function in response to *H. pylori*.^221^ From the aspect of molecular mechanism, *H. pylori* can induce aberrant DNA methylation and downregulate the expression of genes involved in signal transduction pathways and tumor suppression.^222^ Previous studies have found that *H. pylori* infection induces DNA hypermethylation in the promoter regions of upstream-stimulated transcription factor genes *USF1* and *USF2*, and inhibits their expression, which accompanies the development of gastric precancer.^223^ These transcriptional factors may act as tumor suppressors by regulating genes involved in stress and immune responses, inflammation, cell cycle control, and genome stability.^224^ USF1 also binds to p53 as UV-induced DNA damage occurs and prevents the interaction between p53 and the E3-ubiquitin ligase HDM2. This results in p53 stabilization and transient cell cycle arrest.^225,226^ In about half of GC patients, USF1 expression is lower in tumor tissue than non-tumor tissue, and 88% of patients with low USF1 expression have *H. pylori* infection.^227^ Low expression of p53 closely correlates to low expression of USF1, and low expression of both is associated with poor prognosis.^227^

### HGF/c-MET signaling pathway

The mesenchymal epidermal transition factor (c-MET), which is encoded by the proto-oncogene *MET*, is a transmembrane receptor expressed on the surface of epithelial and endothelial cells.^228^ c-MET belongs to the receptor tyrosine kinase (RTK) family, and hepatocyte growth factor (HGF) is the specific ligand for c-Met.^229^ The canonical pathway is activated when HGF binds to c-MET, followed by the homodimerization of c-MET and trans-phosphorylation of its intracellular kinase domains.^229^ These changes form a docking site on c-MET that recruits effector molecules, thus triggering the signals that regulate cell survival, proliferation, migration, and morphogenesis.^230^The major downstream signaling pathways include Ras/MAPK, PI3K/AKT (Fig. 2), Wnt/β-catenin, and signal transducer and activator of transcription 3 (STAT3).^230,231^ There are also many distinct mechanisms of HGF-independent activation of c-MET (non-canonical activation), such as the phosphorylation of c-MET mediated by direct binding of des-gamma-carboxyl prothrombin at the intracellular kinase domain^232^ and crosstalk with other signaling pathways.^233^ While the HGF/c-MET pathway has important physiological functions in normal cellular processes, aberrant activation of this pathway is closely associated with tumor invasion and metastasis in many types of epithelial cancers, such as lung, breast, kidney, liver, ovarian, thyroid, and gastrointestinal tract cancers.^234^ Multiple mechanisms, which can be related to canonical or non-canonical activation or both, may be involved, including gene amplification, activating mutations, transcriptional modification, overexpression, enhanced stimulation by autocrine or paracrine HGF, interactions with other active cell surface receptors, and dysregulations under certain environmental conditions such as hypoxia and inflammation.^235,236^

*MET* gene amplification, high c-MET expression, and co-expression of HGF and c-MET have been found to be significant predictive factors for worse prognosis in GC.^237–239^ Although *MET* gene amplification is relatively rare (4–10%) in GC patients,^240^ c-MET protein overexpression has been detected in up to 82% of cases.^241^ This discrepancy may result from detection methods, whether c-MET protein detection based on both membranous and cytoplasmic staining had a more significant correlation with *MET* gene amplification, compared to that only on membranous IHC.^242^ Another important mechanism is the deletion mutation of the *MET* gene at exon 14 (*METex14del* mutation), which leads to delayed ubiquitination and degradation of c-MET protein.^243^ In a study of 230 patient specimens, including 42 GC, 13 tumor samples were found to contain the *METex14del* mutation, among which all had MET overexpression but only one had *MET* gene amplified.^243^ Notably, MET inhibitors inhibit the growth of patient tumor-derived cell lines from GC and colon cancer containing the *METex14del* mutation, suggesting that METex14del can be a potential biomarker for gastrointestinal malignancies.^243^

As an important regulator of many signaling pathways, the HGF/c-Met axis is closely associated with GC development and progression, tumor metastasis, and therapeutic response. Overexpression of c-MET is frequently observed in GC cases with an increased risk of distant metastasis to the liver^244^ or peritoneum.^245^ Recent studies have discovered that the c-MET signaling may be involved in *H. pylori* infection-related GC tumorigenesis and metastasis. Ito et al.^246^ found that both canonical and non-canonical activation of c-MET signaling in GC cells could be promoted by *H. pylori* infection through its virulence factor CagA protein. Furthermore, the phosphorylated active form of c-MET can be secreted in exosomes by *H. pylori*-infected GC cells and transferred to macrophages, which may consequently induce the pro-tumorigenic phenotype conversion of macrophages promoting tumor progression.^247^ Additionally, *H. pylori* infection could increase the intracellular level of heparinase (HPA), an endoglucuronidase found to be carcinomatosis-relevant, leading to the activation of multiple signaling pathways in human GC cells.^248^ Hao and colleagues observed that overexpression of HGF and HPA had a positive correlation with TNM stage, depth of invasion, and poor prognosis in GC patients.^249^ Their further mechanistic study suggested that HGF/c-MET can regulate HPA expression by activating PI3K/AKT and downstream nuclear factor kappa B (NF-κB) signaling. HPA can also mediate the shedding of heparin-binding HGF to enhance HGF liberation, which can jointly induce tumor metastasis.^249^ Therefore, the HGF/c-MET axis and HPA may be effective therapeutic targets for treating *H. pylori*-related GC.

c-MET has been a well-studied target for cancer treatment and numerous targeted inhibitors have been developed. Blocking HGF in cancer-associated mesenchymal stem cells, where HGF is hyper-produced, may also be a potential GC treatment strategy based on a recent in vivo study.^250^ Currently, the precise regulatory cascades of HGF/c-MET in GC cells have not been fully elucidated. Utilizing complimentary deoxyribonucleic acid microarray technology, Koh et al.^251^ identified several downstream molecules of HGF/c-MET signaling, including E-cadherin, urokinase plasminogen activator, and Kisspeptin, which are cell invasion and migration regulators. Moreover, two cell apoptosis modulators, Jun-B and lipocalin-2, are also recognized as interacting with the HGF/c-MET pathway.^251^ Another study demonstrated that the phosphorylation of RhoA, which is a biomarker highly mutated in diffuse GC patients, may be dependent on c-MET activity.^252^ Notably, a c-MET inhibitor prevented GC cell growth only in GC cells transfected with wild-type RhoA but not Y42 mutant RhoA in vivo and in vitro. Thus, the combined levels of c-MET and phosphorylated-RhoA should be used as predictors for prognosis and patient stratification to optimize targeted c-MET therapy.^252^

In addition to downstream effectors, upstream regulators of HGF/c-MET are also important biomarkers and potential targets in GC. The C-X-C motif chemokine ligand 12 (CXCL12) was found to induce interaction of c-MET with caveolin 1 in lipid rafts. This interaction can lead to activation of c-MET, thereby inducing EMT in GC cells and promoting cell migration. Further analysis in clinical samples also revealed a positive correlation between the CXCL12 receptor CXCR4 and c-MET phosphorylation as well as poor patient prognosis, indicating the clinical importance of the crosstalk between c-MET and CXCL12 in GC treatment.^253^ Several miRNAs have been reported to be involved in GC proliferation and metastasis by their regulation of HGF/c-MET expression. It has been reported that miR-1/34a/144/206 directly target the mRNA of c-MET.^254–257^ In contrast, miR-15a/16/195 are found to directly target HGF mRNA.^258^ These are negative regulators of HGF/c-MET expression, which are found down-regulated in GC tumors, implying their potential therapeutic applications to repress HGF/c-MET-mediated cell proliferation and migration in GC. Other in vitro studies have indicated that ETS homologous factor (EHF) may be critical to GC cell proliferation, apoptosis, cell cycle, EMT, and invasion via the activated c-Met pathway,^258^ whereas IL-10 secreted by cancer-associated macrophages (CAMs) may be involved in GC carcinogenesis.^259^ Nevertheless, the clinical significance of miRNAs, EHF, and IL-10 in GC diagnosis and treatment must be further verified.

The HGF/c-MET axis may also be involved in the therapeutic response of GC. In GC cells with HGF/c-MET activation, excessive transphosphorylated c-MET molecules are likely to interact with other receptor tyrosine kinases such as EGFR and HER2 forming heterodimers, which may allow bypass signaling to provoke resistance to corresponding targeted therapies.^260–262^ This provided a clue that co-inhibition of bypassing pathways may be a potential therapeutic application in treating GC. *MET* gene mutations can change the sensitivity of GC cells to targeted drugs by affecting the activation of downstream signaling pathways. Shen et al.^263^ recognized that GC patients carrying *MET* G1163R or D1228Y/N mutations are likely to show resistance to the TKI drug crizotinib, whereas patients with *MET* V1092L, D1228G, or Y1230H mutations could benefit from this targeted therapy. This indicates that MET mutation analysis may be useful for designing precision medication for GC.

### Wnt/β-catenin signaling pathway

The Wnt/β-catenin signaling pathway is involved in cell proliferation, migration, and death, and is important for the development and homeostasis of some tissues.^264–266^ The β-catenin protein is a transcriptional coactivator in Wnt pathway, which has been found to be involved in a number of biological processes of tumor cells, including proliferation,^267,268^ anti-apoptosis,^269^ and infiltration transfer.^270^ The Wnt/β-catenin pathway is activated when the Wnt ligands bind to the seven-transmembrane receptor Frizzled (FZD) and the low-density lipoprotein receptor-related protein 5 or 6 (LRP5/6).^271^ The Wnt-FZD-LRP5/6 trimer complex recruits disheveled (DVL) and axin through the intracellular domains of FZD and LRP5/6, thereby inhibiting β-catenin phosphorylation and ensuring β-catenin stability. β-catenin then detaches from degradation complexes and accumulates in the cytoplasm, enabling the Wnt pathway to promote cancer progression during the cell cycle.^272–274^ Elevated cytoplasmic and nuclear levels of β-catenin promote the cooperation of β-catenin with T cell factor/lymphoid enhancer factor (TCF/LEF) transcription factors to activate the expression of Wnt-responsive genes^275^ **(**Fig. 2**)**. Several mutant component molecules of typical Wnt signaling lead to aberrant activation of the Wnt/β-catenin pathway,^276,277^ which further contributes to the malignant transformation and invasion of GC.^278,279^

Upregulation of Wnt-1 ligands has been shown to promote advanced GC development.^280^ In contrast, Wnt-2 enhancement is closely associated with gastric tumor formation, invasion, or dissemination.^281^ Studies have found that Wnt-5a can stimulate the migration and invasion of GC cells, mainly through the activation of focal adhesion kinase (FAK) and the small GTP-binding protein Rac.^282^ Overall, dysregulation of Wnt/β-catenin signaling is observed in more than half of the patients and is considered a primary mechanism of GC development.^276,283^ Although persistent activation of Wnt/β-catenin signaling is shown to be related to chemoresistance,^284,285^ the mechanism remains largely unexplored. Several researchers found that activation of Wnt/β-catenin signaling can inhibit ferroptosis in GC cells by attenuating the production of intracellular lipid ROS or inducing glutathione peroxidase 4 (Gpx4) expression by the direct binding of β-catenin/transcription factor 7 like 2 (TCF7L2, also known as T cell factor 4, TCF4) transcriptional complex to the promoter region of Gpx4.^286–288^ The latter mechanism was verified by two studies demonstrating that deficiency in TCF4 promoted cisplatin-induced ferroptosis both in vivo and in vitro.^286,289^ Modulating ferroptosis through regulating Wnt/β-catenin signaling may be a potential therapeutic strategy for improving chemosensitivity in advanced GC.^286^ Finally, targeting Wnt/β-catenin signaling may also improve the therapeutic outcomes of radiotherapy and immunotherapy due to the involvement of ferroptosis.^286,290^ A recent study demonstrated that the Wnt/β-catenin signaling pathway is inversely correlated with the infiltration of T cells in the tumor microenvironment (TME), and, as a result, affects the therapeutic efficacy of PD-1 antibodies.^289,291–293^ It has been found that the disruption of the Wnt/β-catenin pathway in GC cells inhibited their migration and invasion.^294^ Meanwhile, down-regulation of Wnt/β-catenin may enhance the sensitivity of GC cells to PD-1 antibody.^295,296^ This result further suggests that jointly targeting to inhibit β-catenin and PD-1 jointly may be a potential and effective treatment for GC patients.

Different mechanisms can facilitate tumor cell survival and proliferation mediated by activated Wnt/β-catenin signaling in GC. β-catenin-activated CCL28, which is a mucosae-associated epithelial chemokine, can regulate T cells in vitro.^297^ In a clinically relevant mouse GC model established by *Helicobacter felis (H. felis)* infection and the carcinogen *N*-methyl-*N*-nitrosourea (MNU), using a Wnt signaling pathway inhibitor iCRT14 to inhibit β-catenin/TCF activity resulted in decreased CCL28 expression and Treg expression in the stomach cell infiltration.^297^ Furthermore, the anti-CCL28 antibody significantly attenuated Treg cell infiltration and tumor progression in the *H. felis*/MNU mouse model.^297^ This study extended the previous understanding of the oncogenic role of the Wnt/β-catenin pathway mainly through its control of cell proliferation, survival, and differentiation in GC, and confirmed that the immunoregulatory function of the β-catenin signaling pathway also plays an important role in tumor progression.^297^ More importantly, CCL28 blockade exhibits a surprising antitumor effect by inhibiting Treg cell infiltration, providing a new idea for the immunotherapy of GC.^297,298^ E-cadherin, a component of the β-catenin degradation complex, also plays a crucial role in negatively regulating Wnt signaling.^299^ β-catenin is in direct contact between cadherin and α-catenin, the latter interacting with the actin cytoskeleton to form tight cell-cell junctions.^299,300^ As cadherin may maintain the activity and function of β-catenin on the membrane during EMT by competing with its degradation mechanism, the ability of β-catenin to bind to cadherin is essential when the transcription proceeded because cadherin may stabilize β-catenin on the membrane by competing with its degradation mechanism during EMT.^301,302^ In brief, the connection between cadherin and β-catenin may be one of the mechanisms of the EMT process in GC,^303^ and may provide new options for GC diagnosis or therapeutic interventions in the future.^304^

### NF-κB signaling pathway

The NF-κB family of transcription factors consists of several members—RelA, RelB, c-Rel, NF-κB1(p50), and NF-κB2(p52)—which form dimers (homo- and hetero-) and modulate the expression of a variety of genes.^305^ The typical dimer refers to the heterodimer of RelA and p50 subunits.^306^ The canonical or classical NF-κB pathway is activated by different receptors, including tumor necrosis factor receptors (TNFRs), Toll-like receptors (TLRs), and interleukin-1 (IL-1R). NF-κB is kept inactive in the cytoplasm bound to members of the IκB family (IκBα, IκBβ, and IκBγ).^307^ Upon stimulation, the IκB kinase (IKK) complex is activated, leading to phosphorylation of IκBα at Ser32 and Ser36 by IκBβ,^308^ followed by poly-ubiquitination and subsequent degradation of IκBα by the 26S proteasome (Fig. 2). Degradation of IκBα sets NF-κB free, and it translocates to the nucleus where it binds to the promoters of downstream target genes, thus promoting GC progression.^309–311^

The NF-κB signaling pathway is one of the most critical cellular signaling pathways and has an important role in apoptosis and cell survival.^312,313^ One of the main functions of NF-κB is regulation of transcription of inflammatory molecules. NF-κB can regulate the expression of many inflammatory mediator genes related to inflammation and immune response, including *bcl-2*, *bcl-xl*, *cIAP*, *BIRC5*, *TRAF*, *COX-2*, *MMP-9*, *iNOS*, and various cell cycle regulators.^314,315^ The NF-κB pathway also plays a key role in EMT and cancer stem cell activities^316^ and has an important role in tumor formation and tumor development through its anti-apoptotic effect. Inhibition of NF-κB signaling can induce apoptosis and cell cycle arrest in GC cells.^317,318^ In tumorigenesis and development, NF-κB is more likely to play a key linking role in signaling pathways. Proto-oncogene mutation affects upstream factors of the NF-κB signaling pathway, and these factors activate the NF-κB signaling pathway and downstream effectors and initiate gastric carcinogenesis.^319^ Uncontrolled NF-κB signals lead to the occurrence of many tumors, and the abnormal activation of NF-κB in tumors may be one of the main anti-apoptotic factors in GC cells.^319,320^ When activated, it can generate strong anti-apoptotic signals and accelerate tumor development.

At the same time, NF-κB can promote tumor formation by a non-apoptotic mechanism, by directly stimulating cell proliferation through the activation of the proto-oncogenes *c-myc*^321^ and *CCND1* (encoding cyclin D1).^322^ As a target gene of NF-κB, *CCND1* transcription initiated by NF-κB promotes the cell cycle transition from G1/G0 phase to the S phase, leading to cell proliferation and transformation into malignant and cancerous cells.^323,324^ NF-κB can also upregulate hypoxia-inducible factor 1 (HIF-1), which initiates gastric carcinogenesis by promoting tumor angiogenesis.^325,326^ Studies have shown that connective tissue growth factor (CTGF) is upregulated in clinical tissue specimens of GC.^327^ In vitro experiments have shown that high expression of CTGF in advanced GC cells significantly increases tumor metastasis, while RNA interference-mediated knockout of CTGF significantly inhibits cell metastasis.^328^ This process demonstrates the promotive effect of CTGF on GC invasion and metastasis via the downregulation of E-cadherin and activation of NF-κB (Fig. 2). Similar studies also found that the expression of proteinase-activated receptor-1 (PAR-1) stimulates NF-κB activation, thereby initiating the invasion and metastasis of GC.^329^ Additionally, it has been found that NF-κB activation is associated with the heparanase gene expression in GC and is significantly correlated with GC invasion-related features such as lymph node invasion, pathological stage, and depth of invasion.^330,331^ Therefore, NF-κB may become a potential therapeutic target for inhibiting GC invasion and metastasis.^324^

The upregulation of the NF-κB signaling pathway is involved not only in the occurrence of tumors but is also associated with chemoresistance and radioresistance.^332,333^ NF-κB inhibitors may enhance the efficacy of antitumor drugs or increase sensitivity. With the improvement of the rapid detection technology of NF-κB activity and the understanding of the mechanism of NF-κB activation, many drugs that inhibit the activation of NF-κB have been developed. Natural drugs targeting NF-κB have exhibited potential as chemotherapy for GC.^334–337^ For example, Ji and colleagues have reported that tetramethylpyraz, a natural alkaloid, induces GC cell apoptosis by downregulating NF-κB and cyclin D1.^338^ Therefore, screening chemotherapeutic drugs with NF-κB-targeting effects may be a potential strategy for improving chemotherapy.

### TGF-β signaling pathway

Transforming growth factor-β (TGF-β) is a family of active polypeptides that are physiologically involved in embryonic growth and development, stem cell differentiation, wound healing, and inflammation regulation.^339^ The secretion disorder of the TGF-β family is closely associated with the development of tumors.^340^ The TGF-β family consists of three forms with similar biological functions: TGF-β1, TGF-β2, and TGF-β3.^340^ Among them, TGF-β1 has the highest expression level.^341,342^ TGF-β1 is a multifunctional cell growth factor and a multi-type cell proliferation inhibitor.^343^ TGF-β1 can inhibit the proliferation and differentiation of various cells by binding to its receptors, such as TGF-β R1.^344^ It is widely involved in cell morphological changes, adhesion, metastasis, and apoptosis.^345,346^ The expression of TGF-β1 and TGF-βR1 is closely related to the biological behavior and prognosis of malignant tumors.^347^ TGF-β1 is the signaling protein of the *DPC4 (SMAD4*) gene, a tumor suppressor gene. The Smad4 proteins, which have an important impact on the occurrence, development, and metastasis of malignant tumors,^348^ are vital downstream effectors of the TGF-β signaling pathway.^349^ TGF-β ligands bind to membrane receptors to form two types of receptor heterodimers, type I and II, which can activate downstream Smad2 and Smad3 proteins and then combine with Smad4 to form a transcription complex in the nucleus, thereby regulating the transcription of target genes and exerting inhibitory effects on cell growth.^340,350^

TGF-β1 is generally considered a negative cell growth regulator and is strongly correlated with the occurrence and progression of GC and its clinicopathological features.^340^ TGF-β1 in normal gastric mucosa is expressed mainly in the cytoplasm of epithelial cells and some mucous cells and in the cytoplasm of cancer cells in GC tissue.^351^ A retrospective study of 50 patients with GC after surgery found that the 5-year survival rate of patients with high TGF-β1 expression was significantly lower than that of patients with low TGF-β1 expression, indicating that the expression of TGF-β1 is closely related to the prognosis of GC patients.^352^ However, depending on the cell type and physiological environment, TGF-β1 can exhibit opposite effects. TGF-β1 has a significant growth inhibitory effect on cells of epithelial origin by preventing cells from the G1-S phase in vitro,^353,354^ and TGF-β1 expression is often reduced or absent in malignant tumors.^355^ TGF-β1 can also inhibit the proliferation and induce apoptosis of GC cell lines HSC-39 and HSC-43 in vitro.^356,357^ However, the results of another study showed that TGF-β1 protein was highly expressed in GC and increased as the differentiation degree decreased, indicating that TGF-β1 may play a role in the malignant transformation and proliferation of tumors.^358^ The high expression of TGF-β1 in GC cells may also be due to the blockade between TGF-β1 and receptors, resulting in an accumulation of TGF-β1;^359,360^ the elevated TGF-β1 level may promote tumor growth rather than inhibit it, but it does not lose its inhibitory effect on immune cells such as NK and LAK, leading to immune escape of cancer cells.^361,362^ Both TGF-β and its receptors are highly expressed in early penetrating GC tissues, which is related to the strong growth and infiltration ability of this type of GC.^363,364^

Moreover, the TGF-β signaling is one of the main inducers of EMT, which may be related to its crosstalk with the AMPK pathway.^350^ AMPK activation not only inhibits the EMT process of GC cells regulated by TGF-β, but also inhibits the production of TGF-β.^365,366^ Smad3 was found to play a key role in these two processes as well. AMPK can inhibit the phosphorylation and the nuclear translocation of Smad3 protein, thus inhibiting the transcriptional regulatory functions of TGF-β.^366,367^ Therefore, inhibiting the phosphorylation of Smad3 may serve as a new therapeutic target for GC.

### Immune checkpoint signaling pathways

The growth and progression of cancer are directly related to the suppression of the immune system, where inhibitory immune checkpoints play a vital role. Immune checkpoints are modulators of the immune system that either promote (co-stimulatory molecules) or stop signaling (co-inhibitory molecules) in immune cells and control their activity, thus, playing a crucial role in maintaining immune homeostasis in immune cells.^368,369^ The first immune checkpoint molecule, cytotoxic T-lymphocyte–associated antigen 4 (CTLA-4), was discovered by Brunet et al. in 1987.^370^ its function was unclear until 1995, when Allison et al. revealed CTLA-4 to be an important immune checkpoint molecule with great potential as a target for cancer therapy.^371^ Immunosuppressive checkpoint molecules, such as PD-1, CTLA-4, T-cell immunoglobulin and mucin-domain containing-3 (TIM-3), Lymphocyte-activation gene 3 (LAG-3), and T cell immunoreceptor with Ig and ITIM domains (TIGIT), are usually expressed on T cells and bind to their ligands on other cells, thereby triggering negative regulations on immune signaling pathways and preventing immune damage.^369,372–375^ In tumor cells, upregulation of ligands of these inhibitory immune checkpoints during tumor progression helps suppress antitumor immune responses and induce tumor immune escape.^369,376^ Therefore, targeting immune checkpoints is a vital approach of immunotherapy in cancer treatment.

**Fig. 3:**
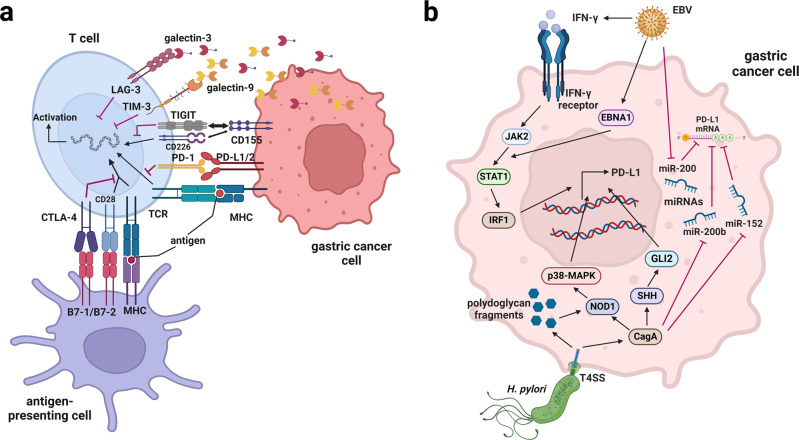
The immune checkpoint signaling pathways in gastric cancer and regulations on PD-L1 by *H. pylori* and EBV. **a** The immune checkpoint proteins PD-1 on the surface of T cells interact with the ligands PD-L1/PD-L2 on GC cells, or the aberrant CTLA-4 proteins on GC patient T cells interact with B7 on antigen-presenting cells, resulting in an immunosuppressive microenvironment, providing cancer cells with a survival advantage. TIGIT on the T cells membrane competes with the activation of CD226 binding to CD155 from the GC cells. Other immune checkpoint proteins, TIM-3 or LAG-3, interact with galectin-9 or galectin-3 released from GC cells, inhibiting the activation of T cells. **b** Chronic *H. pylori* or EBV infection, which are risk factors of GC, can induce upregulation of PD-L1 in GC cells via various signaling pathways and microRNAs, promoting immune escape. EBV Epstein–Barr virus, PD-1 programmed death 1, PD-L1/2 programmed death ligand 1/2, CTLA-4 cytotoxic T-lymphocyte-associated protein 4, TCR T-cell receptor, MHC major histocompatibility complex, TIGIT T cell immunoreceptor with Ig and ITIM domains, TIM-3 T cell immunoglobulin and mucin-domain containing-3, LAG-3 lymphocyte-activation gene 3, IFN-γ interferon gamma, JAK2 Janus kinase 2, STAT1 signal transducer and activator of transcription 1, IRF1 interferon regulatory factor 1, EBNA1 Epstein–Barr nuclear antigen 1, MAPK mitogen-activated protein kinase, NOD1 nucleotide-binding oligomerization domain-containing protein 1, SHH Sonic hedgehog protein, CagA cytotoxin-associated gene A, T4SS type IV secretion system. This figure was created with Biorender.com

Different immune checkpoint molecules and their ligand-receptor signaling are summarized in Fig. 3a. PD-L1 and PD-L2 are transmembrane proteins, which are considered co-suppressors of the immune response. Upon the binding of PD-L1/PD-L2 to PD-1, the proliferation and cytokine secretion of PD-1-positive T cells are reduced, while apoptosis is activated. For cancer cells with PD-L1/PD-L2 expression, attenuating host anti-tumor immune response provides survival advantages for the cancer cells.^377,378^ In the CD28/CTLA-4/B7 co-stimulatory pathway, CD28 is one of the proteins expressed on T cells that produce co-stimulatory signals required for the activation of T cells; CTLA-4 proteins located on T cells function to help keep the body’s immune responses in check; and B7-1/2 are checkpoint proteins on the membrane of activated antigen-presenting cells (APC).^379^ T cells can be activated when the T cell receptor (TCR) binds to the antigen and major histocompatibility complex (MHC) proteins on the APC, accompanied by CD28 binding to B7-1 (CD80) or B7-2 (CD86) on the APC.^380^ However, when B7-1/B7-2 binds to CTLA-4, the T cells are inactivated and unable to kill tumor cells in the body.^381^ Using an immune checkpoint inhibitor (an anti-CTLA-4 antibody) to block the binding of B7-1/B7-2 to CTLA-4 allows the T cells to be activated and kill tumor cells.^382^ The TIM-3/galactin-9 and LAG-3/galactin-3 pathways are similar to the PD-1/PD-L1 pathway. The binding of TIM-3 present on activated T cells to the ligand galactin-9 on tumor cells blocks the response of interferon-γ (IFN-γ) -producing CD4^+^ T helper 1 (Th1) cells and induces apoptosis of CD4^+^ and CD8^+^ T cells, resulting in immune tolerance.^383^ TIM-3 may also be co-expressed with PD-1 in tumor-infiltrating immune cells and act synergistically to mediate effector T cell depletion and dysfunction.^384^ LAG-3 on activated T cells is associated with reduced anti-cancer immune response by inhibiting CD8^+^ T cells upon binding to galactin-3 in tumor cells.^373^ TIGIT is a co-inhibitory receptor that is highly expressed in the tumor-infiltrating lymphocytes in various malignant cancers.^385^ TIGIT can downregulate the immune response either by competing for CD155 ligand binding with CD226 thereby reducing the CD266/CD155-dependent co-stimulation of T cells,^386–388^ or by directly transmitting inhibitory signals to effector cells.^389^ Among these pathways of immune checkpoints, the PD-1/PD-L1 signaling is the most widely studied as a diagnostic/prognostic biomarker as well as a therapeutic target of GC.

Transcriptome analysis of the TCGA subtypes in GC has revealed that immune cell signaling is significantly upregulated in EBV^+^ or MSI subtypes compared to the other two subtypes.^390^ The different levels of immunomodulation shown by the four TCGA subtypes have opened a stratifying strategy for GC patients to maximize immunotherapy efficacy, while immune cell signaling has gained extensive attention in GC research. High content of immune cells, downregulation of genes involved in cytokine/chemokine pathways, and upregulation in PD-L1 and/or PD-L2 expressions are frequently found in EBV^+^ GC cases.^391,392^ In contrast, the MSI subtype is characterized by increased mutation rates and DNA hypermethylation profiles for DNA mismatch repair genes like *MSH1*, *MSH2*, *MSH3*, and *MLH1*, which results in alterations in length with short, repeated DNA sequences (microsatellites) and enhanced expression of neoantigens.^41,393^ Because of the increased neoantigen recognition and the corresponding expression of immune checkpoints in the tumor microenvironment, GC of MSI subtype exhibits high CD8^+^ T cell infiltration and is more sensitive to immune checkpoint inhibitors.^394,395^

Elevated mRNA levels of PD-1, PD-L1, and PD-L2 have been observed in GC patients.^396^ Yun et al.^397^ found that *HER2*, *PD-L1*, and *PD-1* gene expressions in GC are related to staging and lymph node metastasis. The elevated PD-L1 expression is correlated with certain GC molecular subtypes. Liu et al.^398^ observed that PD-L1 was expressed in 59.3% of GC patients and correlated with MSI and EBV^+^ subtypes. *H. pylori*-positive gastric tumors have also been found to have higher PD-L1 expression and T cell hypo-responsiveness, which is considered one of the carcinogenesis mechanisms by *H. pylori* infection.^399^ During GC initiation and progression, chronic EBV or *H. pylori* infection induces immunomodulation from a pro-inflammatory state recruiting immune cell infiltrations to an immunosuppressive microenvironment where PD-L1 is upregulated in GC cells.^400^

However, different mechanisms are involved in EBV- and *H. pylori*-induced PD-L1 upregulation. In EBV-associated GC, the PD-L1 expression on tumor cells is triggered by interferon-γ (IFN-γ) via the JAK2/STAT1/interferon regulatory factor-1(IRF1) signaling pathway.^401^ The EBV nuclear antigen 1 (EBNA1), which is a transcription factor that maintains EBV genome copy number during cell division, may also be a regulator of IFN-γ-induced PD-L1 expression.^401^ Compared to other GC subtypes, EBV-associated GC displays low expression levels of the PD-L1-targeting miR-200 family, which may also contribute to the high expression of PD-L1.^402^

Upregulation of PD-L1 by *H. pylori* in gastric epithelial cells primarily involves the activation of upstream signaling pathways that promote PD-L1 expression. The two major pathways are the nucleotide-binding oligomerization domain-containing protein 1 (NOD1)-dependent activation of p38-MAPK pathway promoted by the *H. pylori* type 4 secretion system (T4SS) components including the effector protein CagA and peptidoglycan fragments,^403^ and the CagA-dependent activation of sonic hedgehog signaling pathway.^404^ Infection by *H. pylori* also negatively affects the expression of PD-L1 suppressor miRNAs, such asmiR-132 and miR-200b, which partially contribute to the elevated PD-L1 expression in *H. pylori*-positive GC^405^ (Fig. 3b). The overexpression of PD-L1 on GC cells inhibits T cell proliferation via the PD-1/PD-L1 inhibitory signaling and induces Treg differentiation from naive T cells, leading to immune escape. Paradoxically, several studies have reported that in advanced GC patients who underwent surgical resection or resection plus adjuvant chemotherapy, the *H. pylori*-positive patients have an improved survival compared to *H. pylori*-negative patients.^406–411^ In a retrospective study involving 49 advanced GC patients, Koizumi et al. observed that the *H. pylori*-positive patients had a significantly better prognosis than *H. pylori*-negative patients in the population of PD-L1-negative, while the prognostic difference was statistically insignificant between *H. pylori*-positive and *H. pylori*-negative patients in the PD-L1-expressing population. The *H. pylori*-positive/PD-L1-negative group showed a potential survival benefit even when the dose of adjuvant S-1 chemotherapy was reduced.^411^ Since the other immune-related parameters, including CD4, CD8, TLC, MMR proteins, and MSI status, did not exhibit a significant correlation with PD-L1 levels or *H. pylori* infection, the immune escape induced by *H. pylori*-dependent PD-L1 upregulation is likely the dominant mechanism of tumor cell survival and poor prognosis.^411^ Therefore, the PD-L1 expression should be taken into consideration when *H. pylori* infection is used as a prognostic factor in GC.

Although PD-L1 overexpression is more likely to be detected in GC with deeper tumor infiltration and lymph node metastasis,^412,413^ PD-L1 can be a positive prognostic biomarker. Detection of PD-L1 or detection of both HER2 and PD-1/PD-L1 in GC may provide a vital reference for stratifying patients who can benefit from checkpoint inhibitor immunotherapy or targeted therapy. As a result, regulatory factors that induce PD-L1 expression have gained attention in developing strategies to increase immunotherapy efficacy. IFN-γ signaling has been shown to be involved in regulating not only the expression level of PD-L1^414^ but also the binding affinity of PD-L2 to PD-1.^415^ Moreover, PD-L1 expression can be stimulated by inhibition of autophagy via the IFN-γ signaling pathway,^414,416^ implying that pharmacological modulation of autophagy may be a novel strategy for improving the efficacy of PD-L1 blockade. On the other hand, miR-105-5p was found as a negative regulator of PD-L1 expression, highlighting it as a potential biomarker for PD-1/PD-L1 immunotherapy and a target for combinational regimen.^417^ However, it should be noted that taking the timing and site of PD-L1 expression into consideration is necessary. Kim and colleagues reported that in the mouse GC model, 5-FU and oxaliplatin reduced the numbers of myeloid-derived suppressor cells to increase the anti-GC efficacy of the PD-1 inhibitor and promote tumor infiltration by CD8^+^ T cells.^418^ However, these chemotherapeutic agents might also mediate induction of PD-L1 expression in tumor cells leading to tumorigenesis of gastric epithelial cells and tumor progression.^418^

Genetic alteration of CTLA-4 in humans has been associated with GC development;^419^ however, CTLA-4 may not be a good target in treating cancer according to the current knowledge. Liu et al.^420^ reported that the association of *CTLA-4* single nucleotide polymorphism with noncardiac GC is not significant in a Chinese population. A recent case report showed hyperprogression of the lymph nodes and liver lesions compressing the gastric stump from a 68-year-old patient with stage IV MSI subtype GC after receiving immunotherapy of durvalumab (PD-1 inhibitor) and tremelimumab (CTLA-4 inhibitor).^421^ More study is still needed to evaluate the therapeutic significance of CTLA-4 in GC.

TIM-3 is an independent indicator of poor prognosis in GC patients and may play an essential role in the progression, invasion, and metastasis of GC.^383,422^ TIM-3 expression is induced on NK cells and tumor-infiltrating T cells during the development of GC, making it a potential indicator for evaluating the tumor progression.^375,423^ Elevated expression of the TIM-3 ligand galectin-9 on cancer cells has been associated with blood vessel invasion and TNM stage in GC.^374^ However, the prognostic value of galectin-9 remains controversial. Long et al.^424^ and Jiang et al.^425^ reported that low expression of galectin-9 in GC patients was associated with poor survival, whereas the study from Wang et al.^374^ reported that galectin-9 expression negatively correlated with poor prognosis in GC patients.^374^ This discrepancy may occur because of differing functions of galectin-9 in different immune states of the patients. As the galectin-9 function remains poorly understood, further research is needed to clarify whether it has a possible tumorigenic role or tumor-suppressing activity. Therefore, TIM-3 is thought to be a relatively promising biomarker and therapeutic target for GC compared to its ligand. In preclinical studies, TIM-3 inhibitors showed similar effects to PD-1 inhibitors, and a combination of PD-1 and TIM-3 inhibitors enhances T cell responsiveness to tumor antigens with synergistic effects, suggesting that TIM-3 may be a useful target in treating GC resistant to anti-PD-1 immunotherapy.^426,427^ The expression of TIM-3 inhibitory ligands on GC cells might also be potential biomarkers for predicting the treatment response of PD-1 mAb.^428^ Targeting PD-1 and TIM-3 combination immunotherapy may have more therapeutic benefit than mono-immunotherapy for GC patients.

LAG-3 expression has a remarkable synergistic effect with PD-1 on promoting the immune escape of GC cells, which suggests it might be a biomarker of poor prognosis.^369^ Galectin-3, the ligand of the LAG-3 inhibitory pathway, was also found to be a potential indicator for poor prognosis in the diffuse type of GC. However, its utility as a prognostic marker may be population-dependent, since overexpression of galectin-3 was highly significant in the North American cohort but not in the Asian cohort.^429^ Targeting both LAG-3 and PD-1 has become an important cancer immunotherapy strategy.^372,430^ However, the understanding of LAG-3’s mechanism in GC is still minimal, and many fundamental questions remain unanswered. Elucidating the mechanism of LAG-3 in more detail should permit a more rational design for LAG-3-dependent immunotherapy.

TIGIT overexpression in the tumor microenvironment has been observed in GC patients, accompanied by upregulation of its ligands, CD155 and CD112, and is associated with immune escape led by CD8^+^ T cell suppression.^431^ In a co-culture system of T and GC cells, the TIGIT expressing peripheral blood CD8^+^ T cells from GC patients exhibited decreased cellular metabolism and impaired cell functions, which were mediated by TIGIT/CD155 signaling and could be reversed by blockade of CD155.^386^ This suggests that the TIGIT/CD155 pathway can be a GC prognostic indicator and a novel immunotherapy target for treating GC. Bioinformatic analysis revealed that epigenetic regulation (majorly methylation) of TIGIT can affect the prognosis and immunotherapeutic responsiveness of GC.^432^ High TIGIT expression can be utilized to identify patients who are likely to be sensitive immunotherapy thereby improving prognosis. On the other hand, TIGIT may be a potential target for designing epigenetic drugs.^433^ Since TIGIT and PD-1 can be highly co-expressed in CD8^+^ T cells,^431^ TIGIT is expected to be a target for potentiating the benefits of anti-PD-1 therapy.

### Other signaling pathways involved in gastric cancer

Many other signaling pathways have been identified to be involved in GC. Briefly reviewed here are recent discoveries of the signaling pathways relevant to fibroblast growth factors and corresponding receptors (FGF and FGFR), signal transducer and activator of transcription 3 (STAT3), hypoxia-inducible factor-1 α (HIF-1α), Hedgehog, and Notch. Alterations of signaling molecules relevant to cell adhesion and cell junction in diffuse-type GC are also discussed here as distinct molecular characterizations from other histological subtypes.

The fibroblast growth factor receptors (FGFR) are transmembrane proteins expressed widely by different cell types. The FGFR family has 4 members, namely FGFR1, FGFR2, FGFR3, and FGFR4. FGFR1 mutations, FGFR2 amplification, and FGFR3 rearrangements are the most common FGFR alterations found in GC.^434^ When bound with fibroblast growth factors (FGF), FGFRs are activated through phosphorylation of the intracellular tyrosine kinase domain, which then activates several important cellular pathways, including the RAS/MAPK, the PIK3CA/AKT/mTOR, and the Janus kinase (JAK) pathways.^435^ Activation of these signaling pathways can affect angiogenesis, cell mitosis, differentiation, proliferation, and invasive processes.^435^ Dysregulation of the FGF-FGFR axis has been thought to contribute to GC carcinogenesis. Overproduction of FGF presumably promotes communication between epithelial and stromal cells in the tumor microenvironment, which is critical for tumorigenesis.^434^ Alterations of the *FGFR* gene are commonly observed in GC patients, which can be a diagnostic biomarker for GC.^436^ In a large cohort of Chinese GC samples, the prevalence of overall FGFR aberrations was 7%.^437^ In another cohort of GC samples, FGFR2 amplification was found in 4.1% of samples.^432^ A small Hong Kong GC cohort study reported that FGF18–FGFR2 signaling could upregulate yes-associated protein 1 (YAP1) oncogene expression by activating the MAPK pathway effector c-Jun.^438^ Cancers that are co-positive for FGFR2, c-Jun, and YAP1 alterations are associated with worse clinical outcomes, indicating the translational potential of FGFR2–c-Jun–YAP1 as a prognostic predictor and therapeutic target for GC.^438^ FGF18 has also been identified as a potential GC prognostic biomarker and therapeutic target, which can be negatively regulated by miR-590-5p to inhibit gastric tumorigenesis.^439^ In addition to tumorigenesis, the FGF-FGFR axis can affect GC invasion and metastasis. Huang et al. reported that upregulation of FGF7/FGFR2 signals can increase the expression of thrombospondin-1, an extracellular glycoprotein responsible for cell–matrix and cell–cell interactions, possibly by activating the PI3K/AKT/mTOR pathway, and finally lead to enhanced GC cell invasion and migration.^440^

STAT3 is known to be an oncogene that is hyperactivated in many types of cancer, including GC.^441^ The STAT3 pathway is activated by the binding of an extracellular cytokine such as IL-6 or an EGF family member such as HGF to the transmembrane cytokine receptor. Binding triggers the dimerization and transphosphorylation of JAKs, which provide docking sites for STAT3 molecules. The JAK dimers mediate phosphorylation of tyrosine 705 of STAT3, and the activated STAT3 is released from the kinase complex and subsequently translocates into the nucleus.^441^ As a transcriptional factor, nuclear STAT3 regulates the gene expression of a wide range of genes that are involved in promoting cancer cell growth, tumor invasion, and chemoresistance.^442,443^ The STAT3 pathway is significantly involved in the tumor progression and metastasis of GC. STAT3 signaling was reported to drive EZH2 epigenetic modification, which is associated with advanced TNM stage and poor prognosis.^444^ Analysis of patient samples revealed that increased survivin and STAT3 expression significantly correlated with concurrent *H. pylori* infection; moreover, their subcellular localizations are key factors influencing GC progression.^445^ Therefore, STAT3 and survivin expressions can be collectively used as potential prognostic biomarkers and therapeutic targets for GC. Additionally, JAK2/STAT3 signaling may play a key role in GC EMT and metastasis induced by IL-6^446^ or mesothelial-mesenchymal transition of GC.^447^ Recent studies on STAT3-related mechanisms in GC have focused on the regulation by miRNA and long non-coding RNA (lncRNA). miRNAs and lncRNAs are potential upstream regulators of STAT that may fulfill their functions as oncogenes or tumor suppressors by influencing STAT3 expression levels in GC cells.^448–451^ Notably, circular RNAs (circRNAs), a non-coding RNA subclass that serves as competitive endogenous sponges for miRNAs, thereby negatively regulating miRNAs,^452^ have been recognized as potential regulators in GC chemoresistance.^453,454^ Deng et al.^448^ recently reported that elevated circVAPA expression was observed in GC tissues compared to normal tissues; moreover, circVAPA may promote cisplatin resistance and tumor progression in GC by modulating miR-125b-5p/STAT3 axis, making it a potential target for GC treatment.

HIF-1α is the pivotal molecule responsible for cell adaptation to hypoxia.^455^ Under hypoxic conditions, the expression of HIF-1α is upregulated and the inhibition on HIF-1α by hydroxylases is relieved due to lack of oxygen. The activated HIF-1α translocates to the nucleus where it acts as a transcription factor exerting stimulatory or inhibitory regulation on the transcription of target genes responsible for metabolism, inflammation, vascular homeostasis, and tumorigenesis.^456^ The HIF-1α signaling pathway has been thought to promote GC progression by mediating tumor cell proliferation, angiogenesis, EMT, therapeutic resistance, and inhibition of cell apoptosis.^457^ HIF-1α expression may be a predictor of poor overall survival for GC patients.^458,459^ The HIF-1α/microRNAs and HIF-1α/lncRNAs axes have been confirmed to play critical roles in GC progression, metastasis, and chemoresistance. Lin et al.^460^ showed that hypoxia-induced HIF-1α/lncRNA-PMAN inhibits ferroptosis of GC cells in peritoneal metastatic GC. Zhao et al. found that HIF-1α/miR-17-5p axis may contribute to the tumor growth and metastasis of GC by negatively regulating programmed cell death 4 (PDCD4).^461^ On the other hand, dysregulated miR-27a,^462^ miR-421,^463^ and lncRNA-PVT1^464^ may be associated with HIF-1α-mediated cisplatin resistance in GC. Other newly identified HIF-1α-regulating downstream molecules that are closely related to GC EMT and metastasis include N-myc downstream-regulated gene 2 (NDRG2),^465^ CXCR4,^466^ liver X receptor α (LXRα),^467^ and RhoE.^468^ The underlying mechanism of HIF-1α-induced angiogenesis in GC may be relevant to the crosstalk between the HIF-1α pathway and the STAT3 pathway or β-catenin/VEGF signaling.^469,470^ HIF-1α has been proven to be a druggable target, and pharmacologic manipulation of HIF-1α is under investigation as a novel therapeutic approach to GC.

The Hedgehog signaling pathway not only plays an essential role in the growth and development of various tissues during embryonic development but is also an important signaling pathway necessary for maintaining the homeostasis of recognized tissues.^471^ The Hedgehog pathway interconnects with Wnt and FGF signaling, which is important during embryogenesis and tissue regeneration.^472,473^ Through aberrant activation of the Hedgehog signaling pathway, the upregulation of sonic hedgehog (SHH) can lead to pathological consequences of multiple types of cancers, such as gastric, esophageal, pancreatic, and prostate cancers.^474^ SHH is expressed in the fundic glands of the human stomach, and is strongly expressed in embryos.^475^ The activation of SHH signaling affects the transcription of cell cycle regulators such as PTCH1, FOXM1, and CCND2, ultimately modulating cell proliferation.^476,477^ PTCH1, an SHH receptor as well as SHH signaling target, is expressed in parietal and mesenchymal cells. High expression levels of SHH and PTCH1 are significantly associated with poor prognosis in GC, and a high expression level of PTCH1 may be associated with GC progression.^478,479^ Another SHH signaling target, FOXL1, is also expressed in mesenchymal cells and may contribute to the functional maturation of the parietal cell lineage.^477^ SHH regulates growth and differentiation within the gastric mucosa through an autocrine loop and FOXL1-mediated epithelial-mesenchymal interaction.^480^ In GC, the upregulation of SHH can indicate an involvement of autocrine signaling loops and epithelial-mesenchymal interactions in the regulation of parietal cell lineage differentiation or maturation.

The Notch signaling pathway is a highly conserved system that regulates the function of multiple cell types and plays a crucial role in cell differentiation, survival, and proliferation. Activation of the Notch signaling pathway has been observed in tumors. Its abnormal activation is involved in direct intercellular communication and plays an essential role in the formation, development, survival, proliferation, invasion, and metastasis of tumors.^481,482^ Notch signaling activation is associated with various cancers and was recently established as a critical pathway regulating gastric stem cell proliferation and differentiation.^483^ Notch induces excessive cell proliferation by upregulating the expression of nuclear transcription factor NF-κB.^484^ It also promotes epithelial cell proliferation and participates in gastric mucosal carcinogenesis. The reduction of Notch1 gene expression can inhibit the proliferation of GC cells and reduce the ability of tumor migration and invasion.^485^ Therefore, it is closely related to the occurrence, development, and metastasis of GC.^485^ Notch2 can upregulate PI3K/AKT signaling pathwayto enhance the invasive ability of GC cells.^486^ In addition to regulating proliferation, the Notch pathway regulates the differentiation of gastric antral epithelial cells, acting in a global manner.^481^ Therefore, the critical molecular differences in somatic versus sinus stem cell differentiation regulated by Notch signaling will be an important area of future research.^482,487^

Cell junction and cell adhesion proteins play key roles in the tumorigenesis of diffused GC. E-cadherin (encoded by *CDH1* gene) is an adhesive junction protein. Germline *CDH1* gene mutation leads to HDGC, while somatic mutation of *CDH1* is also common in sporadic diffused GC.^41^ These findings highlight the key roles of CDH1 in the formation of diffused GC. *CLDN18-ARHGAP* fusions are also common in a subset of diffuse type GC, including GSRCC.^51,488^ *CLDN18* gene encodes Claudin18 protein, a key component of tight junction, which functions to lock adjacent cells together to form a barrier between the external and internal environment.^489^ There are two Claudin18 isoforms, Claudin18.1 and Claudin18.2, which differ in the first exon of the *CLDN18* gene.^490^ Claudin18.2 is mainly expressed by differentiated cells rather than stem cells of the gastric mucosa.^490^ The expression of Claudin18.2 is maintained in a large fraction of GCs. A meta-analysis by Ungureanu et al.^491^ demonstrated that Claudin 18.2 expression was observed in 34.2% of a combined total of 2055 patients in six studies. Xu et al.^52^ reported a high expression rate of Claudin 18.2 in advanced GSRCC patients. In addition, the disruption of cell polarity in GC exposes the Claudin 18.2 epitope on the surface of tumor cells, which makes it an ideal target for therapy to have strong specificity and low toxicity. On the other hand, the ARHGAP family, represented by ARHGAP26, mediates the hydrolysis of GTP in RhoA, leading to RhoA inactivation.^492^ The fusion of *CLDN18* to *ARHGAP* causes ARHGAP over-expression and over-activation and RhoA inactivation. A highly prevalent *RHOA* gene mutation was also found in recent years by large-scale NGS studies of GC.^493^ RhoA is a small GTPase-like RAS and plays a key role in regulating the dynamics of the actin cytoskeleton and cell movement. However, the role of RhoA in regulating carcinogenesis is controversial since it is unclear whether RhoA mutation is loss-of -function or gain-of-function.^494^ The aberrations of *CDH1*, *RHOA*, and *CLDN18-ARHGAP26* are enriched in the GS subset of GC according to TCGA.^41^ Understanding the crosstalk of these three gene aberrations will be key to revealing the mechanisms leading to tumorigenesis in diffused GC.

Another molecule related to cell adhesion is the trophoblast cell surface antigen 2 (Trop2) encoded by the *TACSTD2* (tumor-associated calcium signal transducer 2) gene, which is a transmembrane glycoprotein and calcium signal transducer.^495^ It is structurally related to the epithelial cell adhesion molecule (EpCAM).^495^ Trop2 was initially discovered in trophoblast cells and is expressed in many normal human tissues.^496^ It is involved in embryonic development and implicated in several oncogenic signaling pathways, such as ERK/MAPK and NF-κB pathways.^497,498^ Trop2 has been found to be overexpressed in about half of GC (47–66% according to two studies).^499,500^ Trop2 may induce EMT and metastasis of GC by directly binding to and activating β-catenin, resulting in the accumulation of β-catenin in the nucleus to facilitate GC cell migration and invasion.^501^

The discussed signaling pathways in GC and the identified biomarkers or potential therapeutic targets are summarized in Table 2. Studies on molecular mechanisms have led to a better understanding of how different signaling pathways affect GC tumorigenesis, progression, metastasis, and resistance to therapeutic drugs. These observations will greatly help to identify new targets for anticancer drugs and novel biomarkers of diagnosis, prognosis, as well as personalized treatments for GC patients.

**Table 2:** The roles and functions of signaling pathways in gastric cancer, and the identified biomarkers as well as potential therapeutic targets

Signaling pathways	Roles and functions		Biomarkers and potential therapeutic targets
	Significant roles in GC	Cellular Biological Processes	
MAPK signaling pathway	Prognosis biomarker and related to chemotherapy resistance	Growth, proliferation, differentiation, migration, invasion, metastasis, apoptosis, ROS, cell cycle	CLDN18.2, RTKs, ERK, p-ERK, JNK, p-JNK, p38-MAPKs, p-p38-MAPKs, MEK, p-MEK, RAS, RAF, miR29, miR181c, miR-939, miR-592, lncRNA-MALAT1, lncRNA-CASC2
HER2 signaling pathway	Prognosis biomarker and related to tumor recurrence	Proliferation, differentiation, migration, survival, metastasis, angiogenesis	EGFR, HER2/3/4, ERK, p-ERK, PTEN
PI3K/AKT/mTOR signaling pathway	Diagnosis and prognosis biomarker, related to chemotherapy resistance	Proliferation, survival, migration, invasion, metastasis, cell cycle, apoptosis, angiogenesis	RTKs, PI3K, AKT, p-AKT, mTOR, p-mTOR, PTEN, mTORC1/2, p70S6K1, GSK3, PDK1
P53 signaling pathway	Prognosis biomarker, related to tumor recurrence and chemotherapy resistance	Proliferation, differentiation, metastasis, cell cycle, apoptosis, immune response, inflammation	CDK, RPRM, p21, p16, TP53INP1, USF1/2, miR-17-5p, miR-20a, miR-181a, miR-449, miR-650
HGF/c-MET signaling pathway	Prognosis biomarker, related to chemotherapy resistance	Proliferation, survival, hypoxia, migration, invasion, metastasis, cell cycle, apoptosis, inflammation	RAS, HPA, CXCL12, CXCR4, miR-15a/16/195
Wnt/β-catenin signaling pathway	Diagnosis and prognosis biomarker, related to tumor recurrence and chemotherapy resistance	ROS, proliferation, differentiation, survival, cell cycle, apoptosis, migration, invasion, immune response	TCF4, Gpx4, CCL28
NF-κB signaling pathway	Related to tumor recurrence, chemotherapy resistance and radioresistance	Proliferation, survival, invasion, angiogenesis, metastasis, cell cycle, apoptosis, inflammation	Bcl-2, BIRC5, TRAF, COX-2, MMP-9, iNOS, CCND1
TGF-β signaling pathway	Prognosis biomarker, related to tumor recurrence	Proliferation, differentiation, metastasis, apoptosis, immune response	SMAD, AMPK
PD-1 signaling pathway	Prognosis biomarker, related to immuno tolerance	Proliferation, survival, metastasis, apoptosis, immune response	PD-L1/PD-L2, IFN-γ, miR-105-5p
CD28/CTLA-4/B7 signaling pathway	Immune response		CTLA-4, B7-1/2
TIM-3, LAG-3, TIGIT signaling pathway	Prognosis biomarker, related to tumor recurrence and immune tolerance	Apoptosis, immune response	Galectin-9, galectin-3, CD-155, CD112
FGFR signaling pathway	Diagnosis and prognosis biomarker	Proliferation, differentiation, angiogenesis, migration, invasion, metastasis	RAS, JAK, YAP, miR-590-5p
STAT3 signaling pathway	Diagnosis and prognosis biomarker, related to chemotherapy resistance	Proliferation, invasion, metastasis	IL-6, JAK, EZH2, survivin, miR-125b-5p, miR-143, miR-375, miR-3619-5p, circVAPA
HIF-1α signaling pathway	Related to chemotherapy resistance	Proliferation, survival, angiogenesis, metastasis, cell apoptosis, hypoxia, metabolism, inflammation	NDRG, CXCR4, LXR, RhoE, HIF-1α/microRNAs, HIF-1α/lncRNAs
Hedgehog signaling pathway	Prognosis biomarker	Proliferation, differentiation, cell cycle	PTCH1, FOXM1, CCND2
Notch signaling pathway	Related to tumor recurrence	Proliferation, differentiation, survival, migration, invasion, metastasis	Jagged1, DLL4, Hes1

*MAPK* mitogen-activated protein kinase, *ROS* reactive oxygen species, *CLDN18* Claudin 18, *RTK* receptor tyrosine kinases, *ERK* extracellular signal-regulated kinases, *JNK* c-Jun N-terminal kinases, *MEK* mitogen-activated protein kinase kinase, *RAS* rat sarcoma virus, *RAF* rapidly accelerated fibrosarcoma, *CASC2* cancer susceptibility 2, *EGFR* epidermal growth factor receptor, *HER2/3/4* human epidermal growth factor receptor 2/3/4, *PTEN* phosphatase and tensin homolog, *PI3K* phosphoinositide 3-kinase, *AKT* protein kinase B, *mTOR* mammalian target of rapamycin, *mTORC1/2* mammalian target of rapamycin complex 1/2, *GSK3* glycogen synthase kinase 3, *PDK* pyruvate dehydrogenase kinase, *CDK* cyclin-dependent kinases, *RPRM* reprimo, *TP53* tumor protein p53, *USF1/2* upstream stimulatory factor 1/2, *HPA* human protein atlas, *CXCL12* CXC motif chemokine 12, *CXCR4* CXC chemokine receptor type 4, *TCF4* transcription factor 4, *Gpx4* glutathione peroxidase 4, *CCL28* chemokine ligand 28, *Bcl-2* B-cell lymphoma 2, *BIRC5* baculoviral inhibitor of apoptosis repeat-containing 5, *TRAF* tumor necrosis factor receptor associated factors, *COX-2* prostaglandin-endoperoxide synthase 2, *MMP-9* matrix metallopeptidase 9, *iNOS* cytokine inducible nitric oxide synthases, *CCND1* cyclin D1, *SMAD* suppressor of mothers against decapentaplegic, *AMPK* 5′ adenosine monophosphate-activated protein kinase, *PD-L1/PD-L2* programmed death-ligand 1/2, *IFN-γ* interferon gamma, *CTLA-4* cytotoxic T-lymphocyte-associated protein 4, *TIGIT* T cell immunoreceptor with Ig and ITIM domains, *TIM-3* T cell immunoglobulin and mucin-domain containing-3, *LAG-3* lymphocyte-activation gene 3, *JAK* janus kinase, *YAP* yes-associated protein 1, *IL-6* interleukin 6, *EZH2* enhancer of zeste homolog 2, *VAPA* vesicle-associated membrane protein-associated protein A, *NDRG* N-myc downregulated gene, *LXR* liver X receptor, *RhoE* rho-related guanosine-5′-triphosphate-binding protein, *PTCH1* protein patched homolog 1, *FOXM1* forkhead box protein M1, *CCND2* cyclin D2, *DLL4* delta-like 4, *Hes1* hairy and enhancer of split-1

### Crosstalk between different signaling pathways in gastric cancer

Studies in the emerging field of systems biology have emphasized the complexity of signaling webs during tumor progression. p38-MAPKs activation orchestrates cellular responses by regulating various downstream targets, such as protein kinases and transcription factors, including p53. The functional interaction between p38-MAPKs and p53 appears to occur at multiple levels. The p53 status can directly affect the outcome of p38-MAPKs signaling by negative feedback loops in cells with wild-type p53, altering the biological response of p38-MAPKs activation. Contradictory effects have been reported on the modulation of the p38-MAPKs pathway in cancer. In accordance with its role in p53 activation, it has been proposed that p38-MAPKs activation could act as an onco-suppressive pathway; however, there is also evidence suggesting that p38-MAPK signaling is highly active in various cancer types and promotes tumor growth.^502,503^ The mutant p53 gain-of-function transcriptional target and p38-MAPKs upstream MKK3 and MAP2K have been reported as targets for tumor therapy.^504,505^ In 2021, a study investigating the distinct molecular landscapes of gastroesophageal adenocarcinoma (GEAs) patients with different PD-L1 expression levels identified that tumors with mutations in p53, KRAS, and MAPK pathways were associated with higher PD-L1 combined positive scores (CPSs) in the mismatch repair proficiency and microsatellite stability (pMMR&MSS) subgroup. The data provide potential novel insights for patient selection according to the status of RAS/MAPK pathway alterations and p53 mutations and for the development of rational combination immunotherapies in GEAs.^506^

Hedgehog signaling is important in the regulation of proliferation, survival, and growth of various tissues, including the gastrointestinal tract. Seto et al.^507^ assessed crosstalk between MAPK and hedgehog signaling in the control of cell proliferation in GC. The immunohistochemistry (IHC) results of 35 GC samples suggested that PTCH expression was significantly associated with ERK1/2 phosphorylation as well as SHH expression. The RAS/MEK/ERK signaling cascade positively regulates the transcriptional activity of glioma-associated oncogene homolog 1 (GLI1), a nuclear mediator of the Hedgehog pathway, thereby inducing the expression of hedgehog target genes in GC cells.^508^ Jayati et al. found that hedgehog signaling contributes to inducing PD-L1 expression in GC, and PD-1/PD-L1 inhibition reverses GLI2-induced tolerance, such that combined inhibition of hedgehog signaling and immune checkpoints may be suitable for selected patients.^509^

PD-1/PD-L1 signaling is regulated by various pathways. In gastrointestinal stromal tumors (GIST), knockdown of PD-L1 inhibited the expression level of PI3K, p-PI3K, and p-AKT, whereas the alteration of PI3K/AKT/mTOR pathway blocked PD-1/PD-L1 and attenuated apoptosis of CD8^+^ T cells.^510^ Activation of the PI3K/AKT pathway mediates PD-L1-induced P-gp upregulation in GC drug resistance.^511^ Wang et al.^416^ reported that autophagy inhibition increased PD-L1 expression by increasing the p62/SQSTM1 level and activating nuclear NF-κB in GC, which can be abolished by p62/SQSTM1 inhibition or NF-κB knock down.

The extensive crosstalk between TGF-β signaling and other pathways is a perennial theme of TGF-β research. Several studies have shown that HER2 signaling interplays intimately with TGF-β/Smad in regulating mammary epithelial cell biology and breast cancer progression.^512,513^ The synergy between the TGF-β and HER2/RAS/MAPK signaling can induce the secretion of additional growth factors and cytokines, including TGF-β itself, which in turn induce EMT and tumor invasion.^514,515^ Wnt signaling benefits from extensive crosstalk with other signaling pathways, particularly TGF-β/bone morphogenic protein (BMP) signaling. Wnt and TGF-β signaling often interact to ensure normal tissue homeostasis by modulating the expression of main target genes, and aberrant signaling conduction in either pathway usually results in tumorigenesis. Lei et al.^516^ found that Wnt and TGF-β synergized in the transcriptional activation of the Wnt target gene encoding gastrin, a promoter of GC, indicating that Wnt and TGF-β signaling can cooperate to induce tumorigenesis. Furthermore, the level of Wnt pathway activation inversely associates with the level of Hedgehog pathway activation in gastric tissues. Yanai et al.^517^ demonstrated that the overexpression of glioma-associated oncogene homolog 1 (GLI1), the nuclear mediator of Hedgehog signaling, could restrain Wnt transcriptional activity, nuclear β-catenin accumulation, and proliferation of human GC cells. Referencing this crosstalk between Wnt and Hedgehog pathways may be valuable in developing targeted therapy for GC.

The crosstalk of the STAT3 pathway with other tumorigenic pathways also plays an important role in GC development. In *MET*-unamplified GC, HGF derived from cancer-associated fibroblasts (CAFs) promoted tumor proliferation, migration, and invasion via the activation of the HGF/STAT3/twist1 pathway. CAFs-derived HGF can activate IL-6/STAT3/twist1 pathway by upregulating the expression of the IL-6 receptor.^518^ Additionally, in vivo experiments revealed that HGF from CAFs promoted tumorigenesis and metastasis of *MET*-unamplified GC.^518^ STAT3/c‐Myc and mTOR/pyruvate kinase isozyme 2 (PKM2) signaling pathways were upregulated in human GC. Knockdown of c‐Myc in GC cells downregulated cell proliferation, and knockdown of both PKM2 and c‐Myc were more inhibitory in GC cells than knockout of c‐Myc or PKM2 alone. These observations indicate that co-inhibiting PKM2 and c‐Myc might better antagonize the malignant behavior of GC and c‐Myc might be considered a potential therapeutic target for GC.^519^

Studies have also investigated the crosstalk between downstream pathways of integrin and EGFR. By blocking the synthesis of FAK they detected the effect of crosstalk between EGFR and integrin signal pathways on the proliferation and invasion in a GC cell line, SGC7901, and proved FAK to be a key cross point of two signaling pathways, which makes it a more effective molecular target for GC therapy.^520^

### Epigenetic modifications involved in different signaling pathways of GC

Epigenetic alterations refer to the mechanisms of heritable and reversible regulations on gene expression without changing genomic DNA sequence. Epigenetic modifications include DNA methylation, histone post-translational modification, chromatin remodeling, and change in non-coding RNAs expression. In the past two decades, many studies have highlighted the active roles of epigenetic dysregulations in GC initiation and development. Targeting epigenetic regulators, including the non-coding RNAs, regulatory genes, and the enzymes involved in DNA methylation and histone modification--DNA methyltransferases (DNMTs) and histone deacetylases (HDACs), could be a potential therapeutic approach.^521^

DNA methylation is the transfer of a methyl group from the cofactor S-adenosylmethionine to the C5 position of a cytosine within CpG islands, which are regions with repeated CG dinucleotide sequences located at the promotors of most genes. DNA methylation results in inhibition of gene expression.^522^ Under the TCGA classification, EBV-positive and MSI subtypes of GC tumors generally exhibit a CpG island methylator phenotype (CIMP) characterized by high DNA methylation levels at multiple loci, particularly the tumor suppressor genes.^41^ The CIMP may also be associated with *H. pylori* infection.^523^ In contrast, other GC subtypes may exhibit global hypomethylation associated with proto-oncogene activation and genomic instability.^524^ Alteration of DNA methylation is considered to be an early event of GC tumorigenesis, which mostly occurs in genes that regulate cell cycle (such as *CDKN2A*, *CDKN1B*, *TP53, SMAD2*), DNA repair (such as MLH1, *MSH2*), cell adherence (such as *CDH1*), and cell death (such as *HRAS*).^524,525^ Hypermethylation of *CDH1* promotor plays a vital role in HDGC and is frequently found to accompany *CDH1* mutations or loss of heterozygosity as a second hit to inactivate *CDH1*.^526^ Aberrant methylation also affects genes involved in cancer-related pathways. For instance, hypermethylation of the *DKK3* gene, which is an inhibitory regulator of β-catenin, is commonly found in GC patients inducing activation of Wnt/β-catenin and poor survival.^527^ Hypermethylation of the tumor suppressor gene *ADAMTS9* in GC associates with abnormal activation of the AKT/mTOR pathway and cancer progression.^528^

The post-translational modifications of histone, such as acetylation, methylation, ubiquitination, phosphorylation, and SUMOylation, are important epigenetic mechanisms for regulating chromatin structure and gene expression.^529^ Histone modification plays an important role in GC development relevant to overexpression of oncogenes or downregulation of tumor suppressor genes. Elevated expression of histone deacetylating enzymes HDAC1 and HDAC2 has been observed in human GC tissue samples, and correlates with TNM staging and chemoresistance.^530^ Aberrant upregulation of HDACs is associated with hypoacetylation of histone, which can lead to downregulation of tumor suppressor genes. Reduced acetylation levels of histone H3 and H4 have been suggested to be associated with p21 downregulation and GC progression.^531,532^ Additionally, dysregulation of histone methylation and acetylation is involved in the progression and EMT of GC by cooperative regulation with PI3K/AKT and Wnt signaling pathways.^533,534^

Chromatin remodeling is induced by histone modification and influences the interaction between chromatin-modifying proteins and DNA.^535^ Recent studies have shown that members of the SWItch/Sucrose Non-Fermentable (SWI/SNF) chromatin remodeling complex family can function as tumor suppressor genes. A well-studied example is the *ARID1A* gene. Mutations or deletions of the *ARID1A* gene have been detected in 8-25% of GC and are associated with concurrent gain-of-function mutations of *PIK3CA* and microsatellite instability.^536,537^ Another study by Zhang and colleagues revealed that ARID1A may function as a suppressor of GC cell proliferation by modulating PI3K/AKT pathway via targeting *PIK3CA* and *PDK1*. This provides a novel strategy of using PI3K and AKT inhibitors to treat GC with PI3K and AKT overexpression due to loss or deficiency of *ARID1A*.^538^

Noncoding RNAs (ncRNAs) include lncRNAs, miRNAs, siRNAs, and PIWI-interacting RNAs (piRNAs). The regulatory, potential diagnostic, and therapeutic values of certain lncRNAs, miRNAs, and siRNAs have been discussed in the previous sections or specific signaling pathways. piRNAs are a class of ncRNAs that form complexes with PIWI nuclear proteins to cause histone modifications. Research on the role of piRNAs in GC is still limited. Several studies have shown differential piRNA expression profiles in tumors compared to non-tumor tissues, suggesting that piRNAs can be novel cancer biomarkers. Cheng et al.^539^ reported that piR-651 was overexpressed in human GC cells compared to normal gastric epithelial cells, and individuals at advanced GC stages had higher expression than those at earlier stages. Furthermore, restrained growth of two GC cell lines was observed after inhibition of piR-651, suggesting a potential therapeutic value for targeting piR-651. In contrast, piR-823 expression was found to negatively correlate with GC progression, indicating its tumor-suppressing function.^540^ There have been reports that the piRNA/PIWI complex regulates STAT and AKT pathways in colorectal cancer and liver cancer;^541^ however, these interactions have not yet been reported in GC.

Interplay among different epigenetic mechanisms should be considered in GC. DNA methylation and miRNAs are involved in regulatory feedback loops, while siRNAs and piRNAs can regulate both DNA methylation and histone modification. LncRNAs are regulated by DNA methylation yet can regulate DNA methylation. During this process, some lncRNAs interact with miRNAs,^542^ and the lncRNA-miRNA-mRNA pathway undergoes another epigenetic regulatory step before altering target genes in GC tissues.^543^ A deeper understanding is needed to establish the foundation for designing dual or multiple epigenetic-targeting strategies for GC treatment.

## Progress in therapies for gastric cancer

### Current therapies for gastric cancer

**Fig. 4:**
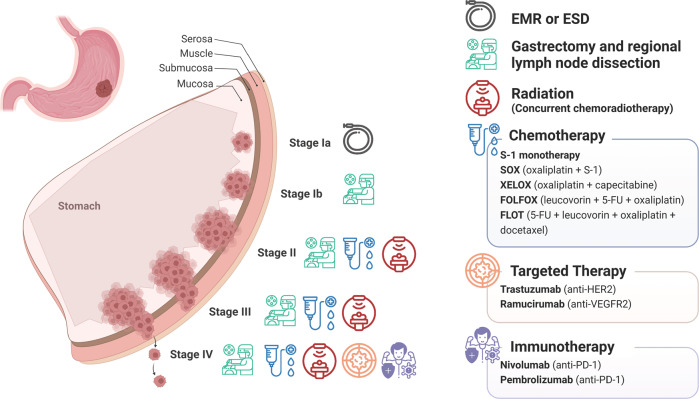
Current therapies for gastric cancer based on staging. Therapeutic interventions for GC at different stages are illustrated by icons. The majorly used drugs or regimens of chemotherapy, targeted therapy, and immunotherapy are listed. EMR endoscopic mucosal resection, ESD endoscopic submucosal dissection. S-1 is an oral agent that is converted to 5-FU in the body, which contains a 5-FU prodrug called tegafur and the two enzyme inhibitors 5-chloro-2,4-dihydroxypyridine (CDHP) and oteracil potassium (Oxo), in a molar ratio of 1:0.4:1. This figure was adapted and modified from “Gastric Cancer Staging” by Biorender.com (2022). Retrieved from https://app.biorender.com/biorender-templates. Icons were adapted from Adobe Express

Even as chemotherapy, radiation therapy, targeted therapy, immunotherapy, and other treatment modalities continue to advance, surgery remains the only radical treatment for GC. The goal of the procedure is to accomplish radical resection, which means that the relevant local lymph nodes are eliminated, and the cutting edge is tumor-free. The two most common surgical procedures are distal gastrectomy and anastomosis of the esophagus with the small intestine after total gastrectomy.^544,545^ The type of procedure for patients who are surgical candidates depends on the various clinical TNM (cTNM) stages of the tumor^28^ (Fig. 4). According to the patient’s physical state, individualized care is required for patients who are unable to undergo surgery.

However, two studies have shown that perioperative treatment, which contains preoperative neoadjuvant therapy and postoperative adjuvant chemotherapy, can effectively improve the 5-year survival rate of GC patients.^546,547^ Preoperative neoadjuvant therapy not only has good safety, but also significantly improves the tumor remission rate, R0 resection rate, and 5-year survival rate without raising the risk of postoperative complications or mortality, according to the results of the RESOLVE and PRODIGY clinical trials.^548,549^ Additionally, the outcomes of two clinical trials, JACCROGC07 and ARTIST-II, demonstrate that postoperative adjuvant chemotherapy can induce positive tumor responses, lower the rate of tumor recurrence and metastasis, and improve the disease-free survival rate (DFS).^548,550^

For stage I GC, endoscopic resection, which comprises endoscopic mucosal resection (EMR) and endoscopic submucosal dissection (ESD), has demonstrated success for treating early GC and is thus the primary option unless there is a significant risk factor, such as lymph node metastasis.^551^ The criteria for EMR and ESD have been expanded to include macroscopically intramucosal (cT1a) differentiated carcinomas >2 cm without ulcer and ≤3 cm with ulcer, and there is no appreciable difference in long-term survival, according to the findings of a multicenter, prospective single-arm research (JCOG0607) in Japan.^552^ EMR and ESD are indicated for intramucosal carcinoma with a diameter of <2 cm, differentiated type, and no ulcer. ESD is indicated for either intramucosal differentiated carcinoma with a diameter >2 cm and no ulcer, or intramucosal differentiated carcinoma with a diameter <3 cm and with ulcer.^552^

For patients who do not meet the criteria for either EMR or ESD, gastrectomy combined with regional lymph node dissection D1 or D2 can be performed by laparotomy or laparoscopy.^553^ All perigastric lymph nodes and left gastric artery lymph nodes, which have the highest risk of metastatic GC, are included in the scope of lymph node dissection D1.^554^ Lymph nodes along the common and proper hepatic arteries, the splenic hilum, and the splenic artery are all included in the scope of the lymph node dissection D2.^554^ According to a Taiwanese randomized clinical study, patients who underwent gastrectomy combined with lymph node dissection D2 had a greater chance of survival than those who underwent gastrectomy combined with lymph node dissection D1.^555^ To increase the precision of staging and prognosis, lymph node dissection requires at least 16 lymph nodes.^556^

Stage II GC is often treated with laparoscopic gastrectomy combined with lymph node dissection D2.^557^ Laparoscopic surgery has emerged as a superior option to the traditional laparotomy method. Laparoscopic surgery has been shown to be safe compared to traditional laparotomy, making it suitable for use as a standard surgical practice, according to the findings of the large-scale prospective investigations JCOG0912 and KLASS01 from Japan and Korea.^558,559^ To improve the tumor remission rate, adjuvant chemotherapy with XELOX (oxaliplatin plus capecitabine) or S-1 monotherapy regimens are needed postoperatively.^557^ Because multiple randomized controlled clinical trials have demonstrated that increasing radiation therapy does not increase overall survival (OS) rates following gastrectomy, postoperative radiation therapy is not advised.^560^

For Stage III advanced GC, the results of two phase III prospective randomized controlled clinical trials, CLASS01 and KLASS02, show that laparoscopic distal gastrectomy combined with D2 lymph node dissection is safer than traditional laparotomy, and reduces intraoperative blood loss, speeds up the recovery of gastrointestinal function, and reduces patient hospitalization time, with no appreciable difference in long-term survival.^561,562^ Preoperative neoadjuvant chemotherapy or chemoradiotherapy, and postoperative adjuvant chemotherapy are important for patients with advanced GC.^557^ Preoperative neoadjuvant chemotherapy can be administered using a number of regimens, including the SOX regimen (oxaliplatin plus S-1),^563^ XELOX (oxaliplatin plus capecitabine), FOLFOX (leucovorin plus fluorouracil plus oxaliplatin), and FLOT (fluorouracil plus leucovorin, oxaliplatin and docetaxel) regimens.^564–566^ DT45~50.4Gy coupled with platinum or paclitaxel is used in preoperative neoadjuvant chemoradiotherapy.^567^ In most cases, XELOX (oxaliplatin plus capecitabine) or SOX (oxaliplatin plus S-1) are used for postoperative adjuvant chemotherapy.^563,564^

Comprehensive therapy is required depending on the patient’s condition for locally advanced, unresectable GC.^568^ Concurrent chemoradiotherapy has been shown in several trials to be more successful than conventional chemotherapy or radiotherapy in reducing the tumor resection rate and increasing the remission rate when the patient is normally in excellent health, and can prolong the survival time of patients.^569^ There are three types of concurrent chemoradiotherapy: (1) DT45~50.4 Gy coupled with carboplatin and paclitaxel; (2) DT45~50.4 Gy coupled with cisplatin or oxaliplatin and 5-FU or capecitabine; and (3) DT45~50.4 Gy coupled with paclitaxel and 5-FU or capecitabine.^567,570,571^ However, chemotherapy or radiotherapy alone can be used if the tumor has spread to numerous lymph nodes and the patient might not tolerate concurrent chemoradiotherapy.^572^ Patients’ clinical symptoms, such as pain relief and bleeding reduction, as well as their quality of life, can be improved by radiotherapy.^573^ Chemotherapy alone can increase the survival rate of patients with poor overall health condition.^574^ Currently, 5-FU, cisplatin, oxaliplatin, paclitaxel, and irinotecan are the most widely utilized chemotherapy medicines. A phase III clinical trial revealed that the combination drug’s effective rate and median OS were dramatically increased.^575^

For Stage IV GC, only systemic antineoplastic medications can be utilized to extend patients’ lives at this point, because surgery is no longer an option due to the organ metastases of cancer cells.^576^ Chemotherapeutic medicines, molecular-targeted therapies, and immune checkpoint inhibitors are now the most widely utilized systemic antineoplastic medications. Trastuzumab,^40^ an anti-HER2 medicine, and ramucirumab, an anti-angiogenesis pathway drug, are the two regularly used molecular-targeted medications. The results of two clinical studies, REGARD and RAINBOW, demonstrated that patients receiving ramucirumab had a longer median survival time and OS rate.^577,578^ In addition, an immune checkpoint inhibitor PD-1 monoclonal antibody, such as nivolumab, can be used in the treatment of refractory cancer.^46^ In comparison to patients who merely received a placebo and supportive therapy, participants treated with nivolumab had a better OS rate, according to a Phase III randomized study ATTRACTION-2.^579^

Additionally, supportive care is crucial in the treatment of advanced GC since it can considerably increase patients’ nutritional and psychological status as well as their survival time.^580^

### Advances in targeted therapy and immunotherapy for gastric cancer

Currently, the development of new drugs for GC focuses on targeted therapy and immunotherapy. Although molecular and cellular evidence suggests many different genes and signaling pathways play key roles in the initiation and progression of gastric cancer, only a fraction is druggable. The current druggable targets reflect the importance of the EGFR/HER2 and c-MET pathways associated with cell growth, the immune checkpoint pathways associated with immune escape, and the cell adhesion and cell junction signaling associated with invasion and metastasis. The most successful target in GC is HER2, which transduces growth signaling and induces proliferation, motility, and invasion of cells. The introduction of immune checkpoint inhibitors, mainly PD-1 antibodies also changed the scheme of GC treatment significantly. Other druggable targets in GC are growth factor receptors, such as EGFR, VEGFR, c-MET, and FGFR2, and enzymes involved in epigenetic regulations like DNMT and HDAC. In addition, a few membrane proteins that are overexpressed in GC cells, including Claudin18.2, Trop2, and Mucin 17 (MUC17), are also targeted by strategies such as antibodies, ADC, bi-specific antibodies, or CAR-T. These drugs are under fast clinical development, which may change the picture of GC treatment in the next few years.

#### HER2-targeted therapies

Drugs targeting HER2, including antibodies, antibody-drug conjugates (ADC), and small-molecule tyrosine kinase inhibitors, are being developed for cancer treatment. The monoclonal antibody trastuzumab was the first agent developed for HER2 targeting and can improve outcomes among women with HER2-positive breast cancer.^581^ In GC, the addition of trastuzumab to standard chemotherapy of HER2-positive GC may increase the survival of the patients.^40^

Although widely used, treatment with the HER2 antibody failed to maintain the control of the tumor, and drug resistance eventually developed. HER2 ADC was developed to further enhance the cytotoxicity of HER2 antibodies. Trastuzumab deruxtecan (DS-8201) is an ADC consisting of an anti-HER2 antibody with the same amino acid sequence as trastuzumab, a cleavable tetrapeptide-based linker, and a cytotoxic topoisomerase I inhibitor exatecan. In a phase II trial, treatment with DS-8201 led to significantly improved response and OS, in comparison to standard chemotherapy, among patients with HER2-positive pretreated GC.^582^ Disitamab vedotin (RC48) is another anti-HER2 ADC containing hertuzumab coupling monomethyl auristatin E (MMAE) by a cleavable linker. In phase II single-arm trial, disitamab vedotin showed promising activity with manageable safety in patients with advanced gastric or gastroesophageal junction cancer overexpressing HER2.^583,584^

Zanidatamab (ZW25) is a bi-specific antibody directed against the two HER2 domains targeted by trastuzumab and pertuzumab, respectively. Zanidatamab was evaluated in phase I study (NCT02892123) in heavily pretreated gastroesophageal adenocarcinoma patients (including prior HER2-targeted therapy). Zanidatamab is well tolerated with promising and durable anti-tumor activity, both as a single agent and in combination with chemotherapy, which may be a good candidate drug for trastuzumab-resistant GC.^585^

Small-molecule tyrosine kinase inhibitors targeting HER2 are also under development for GC treatment. Lapatinib, the first dual inhibitor of EGFR and HER2, was approved by the US FDA in 2007. It is suggested for use in combination with chemotherapy for the treatment of HER2 overexpressing breast cancer.^586^ In the phase III TRIO-013/LOGiC trial, lapatinib was tested in combination with chemotherapy in HER2-positive gastric and esophageal cancer. Unfortunately, the addition of lapatinib to chemotherapy did not increase OS.^587^ In another study, the combination of lapatinib with perioperative chemotherapy for resectable HER2-positive gastroesophageal adenocarcinoma did not improve response.^588^

#### EGFR-targeted therapies

Like HER2, EGFR also plays a key role in various cancer types. Unlike HER2, EGFR is mainly activated through mutations rather than gene amplification. *EGFR* gene mutations, including point mutations and exon 20 insertions, are driver mutations in non-small cell lung cancer (NSCLC). However, *EGFR* mutations in other tumor types including GC are much rarer, and their clinical significance is unclear. Cetuximab, a monoclonal antibody targeting EGFR, is effective in treating colorectal cancer. However, the addition of cetuximab to standard chemotherapy failed to show any improvement in the survival of GC patients in the phase III EXPAND trial.^589^ This study was performed in GC patients not selected by EGFR status, which may be the reason for its failure. Another EGFR antibody, panitumumab, also failed in the phase III trial in unselected GC patients.^590^ Learning from these results, researchers tested the anti-EGFR treatment in EGFR-amplified GC patients. In an early study, researchers identified 19 gastroesophageal cancers with EGFR amplification out of 363 screened patients (5%). The addition of cetuximab to chemotherapy in this small group of patients resulted in high tumor response rates.^591^ Thus, anti-EGFR may be effective in meticulously selected GC patients. More clinical trials are needed to prove this preliminary result.

#### VEGFR-targeted therapies

Blocking angiogenesis has been attempted in GC treatment with varied results. Angiogenesis is predominately regulated by VEGF/VEGFR signaling.^592^ Strategies for blocking angiogenesis signaling include neutralizing VEGF with antibodies, blocking VEGF receptors with antibodies, and inhibiting VEGF intracellular activities with small-molecule tyrosine kinase inhibitors. Unfortunately, targeting VEGF in GC has been unsuccessful. In the phase III AVAGAST study, bevacizumab, a monoclonal antibody against VEGF, was tested as first-line therapy in advanced GC. The combination of bevacizumab with chemotherapy failed to improve the OS of the patients; however, bevacizumab treatment was associated with increases in progression-free survival and overall response rate.^593^

Targeting VEGFR has achieved positive results in GC. In the phase III REGARD trial, the VEGFR2 antibody ramucirumab was tested in advanced gastric or gastroesophageal junction cancer. Ramucirumab monotherapy showed survival benefits in patients.^577^ Apatinib is a selective VEGFR2 small molecule tyrosine kinase inhibitor approved in China.^594^ Phase III clinical trial showed that apatinib monotherapy can increase the OS of repeatedly treated GC patients.^595^ Lenvatinib and regorafenib are multikinase inhibitors with anti-VEGFR activity. These drugs are currently being tested in combination with immune checkpoint inhibitors to treat GC in early clinical trials. Some positive initial results have been observed and the final efficacy needs to be confirmed in larger clinical trials.^596,597^

#### c-MET-targeted therapies

Rilotumumab is a monoclonal antibody targeting c-MET. In the phase II trial, rilotumumab showed some anti-tumor efficacy in gastric and gastroesophageal cancer.^598^ Unfortunately, in the pivotal phase III RILOMET-1 trial, the addition of rilotumumab to chemotherapy failed to improve the outcome of gastric and gastroesophageal cancer.^599^ Currently, research on c-MET inhibitor drugs mainly focuses on tyrosine kinase inhibitors. Savolitinib is a selective c-MET tyrosine kinase inhibitor that was granted approval in China for the treatment of metastatic NSCLC with *MET* exon 14-skipping alterations.^600^ In the VIKTORY umbrella trial, patients with metastatic GC were assigned to eight different biomarker groups to receive corresponding targeted drugs as second-line treatment.^601^ Savolitinib was assigned to treat patients with *MET* amplification. The overall response rate was 50% (10/20). The biomarker-assigned treatment cohort had encouraging response and survival rates when compared to conventional second-line chemotherapy.^601^

#### FGFR2-targeted therapies

There are two main strategies to target FGFRs: using TKIs or antibodies. AZD4547 (ABSK091) is an FGFR1/2/3 inhibitor. The phase II SHINE trial compared AZD4547 with paclitaxel as second-line treatment for FGFR2 amplified metastatic GC. Unfortunately, the trial failed to show improved outcome for those patients.^602^

Bemarituzumab is a first-in-class monoclonal antibody that selectively binds to FGFR2b, blocking ligand binding and induces antibody-dependent cell-mediated cytotoxicity (ADCC). The phase II FIGHT trial investigated the efficacy of bemarituzumab in the first-line treatment for metastatic gastric and gastroesophageal cancer patients. The addition of bemarituzumab to chemotherapy led to a 2-month improvement in progression-free survival (PFS) but failed to extend the OS. The duration of response was longer in patients with higher FGFR2b expression.^603^ This study indicates that bemarituzumab may be used for the first-line treatment of GC.

#### Claudin18.2-targeted therapies

Currently, different strategies are used to target Claudin18.2, including monoclonal antibodies, bispecific antibodies, CAR-T, and ADCs. Zolbetuximab (IMAB362) is a Claudin18.2 targeted antibody. The FAST study enrolled advanced gastric, gastroesophageal junction, and esophageal adenocarcinoma patients.^604^ The addition of zolbetuximab to chemotherapy can improve both PFS and OS. In addition, the side effects were manageable. The combinination of zolbetuximab and chemotherapy was generally tolerated. Zolbetuximab is currently being evaluated in phase III trials (NCT03653507, NCT03504397).

This initial success has attracted more attention to strategies that target Claudin18.2, especially CAR-T. CT041 is a Claudin18.2 targeted CAR-T drug. In phase I of a clinical trial in patients with previously treated digestive system cancers, CT041 showed an acceptable safety profile and encouraging overall response rate (ORR), as well as a 6-month overall survival rate. These initial results suggest that CT041 has promising efficacy in treating GC.^605^

#### Trop2-targeted therapies

Sacituzumab govitecan, the first-in-class anti-Trop2 antibody-drug conjugate (ADC), was approved by the US FDA in 2020 for the third-line treatment of metastatic triple-negative breast cancer (TNBC).^606^ Clinical trials are underway to expand the use of sacituzumab govitecan in multiple solid tumors, including GC. In the phase I/II IMMU-132-01 basket trial, sacituzumab govitecan was tested in refractory metastatic epithelial cancers.^607^ Efficacy was seen in several cancer cohorts, which suggests Trop-2 might be a broad target in solid tumors. Unfortunately, only five GC patients were included in this study and efficacy could not be determined. More studies are warranted to validate the efficacy of sacituzumab govitecan in GC.

#### Immune checkpoint-targeted therapies and other immunotherapies

Immunotherapy is a breakthrough in cancer treatment in the last decade. Immunotherapy in GC has also been progressing very rapidly. Cancer immunotherapy mainly comprises checkpoint inhibitors, adoptive immune cell therapy, and cancer vaccine. Checkpoint inhibitors have been approved to treat various types of solid tumors. Other adaptive immune cell therapies and cancer vaccines are still under clinical investigation in solid tumors.

GCs of MSI or EBV^+^ subtype according to TCGA classification are highly immunogenic with high expression of immune checkpoints, which makes them good candidates for cancer immunotherapy.^608^ Currently, PD-1 inhibitors have been successfully applied in GC treatment. The phase III ATTRACTION-2 study evaluated PD-1 inhibitor nivolumab for repeatedly treated advanced-stage gastric and gastroesophageal junction (G/GEJ) cancer.^609^ According to 2-year follow-up results, OS was significantly longer in the nivolumab group regardless of tumor PD-L1 expression.^609^ In the phase III KEYNOTE-062 trial, the PD-1 inhibitor pembrolizumab, alone or in combination with chemotherapy, was tested as first-line therapy in advanced GC. This trial found that pembrolizumab was not inferior to chemotherapy, and fewer adverse events were observed.^610^ Similarly, nivolumab was also tested as a first-line treatment of advanced gastric, gastro-esophageal junction, and esophageal adenocarcinoma in the phase III CheckMate 649 trial.^611^ Nivolumab with chemotherapy, compared to chemotherapy alone, resulted in significant improvements in OS in patients with a PD-L1 CPS of five or more.^611^ The PD-1 inhibitor might also benefit HER2-positive GC. In the phase III KEYNOTE-811 study, pembrolizumab was added to the standard trastuzumab plus chemotherapy for HER2-positive gastric or gastroesophageal junction cancer. According to interim analysis, the addition of pembrolizumab markedly reduces tumor size and significantly improves objective response rate.^612^

CTLA-4 is another important checkpoint. The CTLA-4 inhibitor ipilimumab has been approved in melanoma treatment.^613^ Unfortunately, targeting CTLA-4 in GC has been unsuccessful. In a phase II trial in pretreated late-stage GC, ipilimumab was not superior to supportive care.^614^ New strategies to combine inhibitors of PD-1 and CTLA-4 have also been tried. Cadonilimab (AK104) is a first-in-class PD-1/CTLA-4 bi-specific antibody developed by a Chinese biotech company. It received marketing approval from the National Medical Products Administration (NMPA) of China in 2022 for cervical cancer.^615^ In a phase Ib/II study, AK104 was evaluated in combination with chemotherapy for the first-line treatment of G/GEJ cancer (NCT03852251). AK104 showed promising activity and manageable safety.^616^ A phase III study of AK104 combined with chemotherapy as first-line therapy for G/GEJ cancer is underway (NCT05008783).

LAG-3 is another inhibitory checkpoint, which can be blocked by the antibody relatlimab. The combination of relatlimab and PD-1 antibody nivolumab has been shown to be safe and effective in melanoma.^617^ Relatlimab in combination with nivolumab is currently being tested in a phase II clinical trial for the first-line treatment in patients with G/GEJ cancer (NCT03662659). In another phase Ib study, relatlimab in combination with nivolumab was tested as an induction treatment prior to concurrent chemoradiation in patients with operable E/GEJ cancer (NCT03044613).

Monoclonal antibodies targeting TIGIT can effectively restore T cell function, exerting an anti-cancer effect.^618^ Tiragolumab is a potent TIGIT inhibitor that has entered clinical trials. Study showed that tiragolumab can enhance the effect of the PD-L1 antibody atezolizumab in non-small-cell lung cancer.^619^ Tiragolumab is also being tested in combination with atezolizumab and chemotherapy in a phase II, single-arm study for the first-line treatment of HER2-negative, unresectable, recurrent, or metastatic G/GEJ cancer (NCT04933227).

Adoptive immune cell therapy is another area of immunotherapy undergoing rapid development. CAR-T therapy lies at the center of adoptive immune cell therapy. CAR-T therapy is highly effective in treating hematopoietic tumors, sometimes leading to the complete remission of tumors. Several CAR-T therapies have been approved worldwide so far.^620^ However, CAR-T therapies have been less impressive in treating solid tumors, and no CAR-T therapy has been approved for solid tumors. As discussed earlier, Claudin18.2 targeted CAR-T is in rapid drug development for GC. Tumor vaccines are still in early clinical development, and their potential in cancer therapy needs to be tested vigorously.

#### Development of targeted therapies under preclinical/early clinical investigations

Several other targets are under preclinical or early clinical investigation that hold the potential to change the treatment of GC in the future. For instance, inhibitors for FAK, a non-receptor tyrosine kinase that regulates cell adhesion and cell survival,^621^ are currently under early clinical investigation. Many FAK inhibitors have been tested in various cancer types with disappointing results.^621^ IN10018 is a FAK inhibitor that showed robust efficacy in patients with platinum-resistant recurrent ovarian cancer.^622^ IN10018 is under evaluation in a phase I trial in previously treated locally advanced or metastatic G/GEJ adenocarcinoma (NCT05327231). Interestingly, a recent in vivo study showed that diffuse gastric cancer with RHO-A mutations was specifically sensitive to FAK inhibitor.^494^

Tyrosine receptor kinase (TRK) receptors, encoded by neurotrophic receptor tyrosine kinase (*NTRK*) genes, are predominantly expressed in neuronal tissue. Fusion of *NTRK* genes is a driver mutation;^623^ however, this kind of mutation is rare (<0.4%) in GC. The TRK inhibitor entrectinib is approved in the US and Europe for the treatment of patients with certain types of solid tumors expressing an *NTRK* gene fusion.^624^ GC patients with *NTRK* fusions can also be candidates for NTRK inhibitor therapy,^625^ but the efficacy of TRK inhibitors in treating GC requires further validation.

DKN-01 is a humanized monoclonal antibody that targets the DKK1 protein, which modulates Wnt/β-catenin signaling and is a crucial prognostic factor predicting tumor recurrence and survival in advanced GC patients.^626^ The FDA granted an Orphan Drug Designation to DKN-01 for the treatment of patients with G/GEJ cancer.^627^ DKN-01 is also an immunomodulatory combination partner for the treatment of cancer. In a phase III study, DKN-01 is under evaluation in combination with PD-1 antibody tislelizumab for the treatment of patients with locally advanced or metastatic G/GEJ cancer (the DisTinGuish study; NCT04363801).

AMG 199 is bi-specific antibody targeting CD3 and MUC17 that was designed to engage CD3^+^ T cells to MUC17-positive G/GEJ cancer cells, mediate redirected tumor cell lysis, and induce T cell activation as well as proliferation.^628^ A phase I clinical trial is being conducted to test AMG 199 in patients with MUC17-positive G/GEJ cancer (NCT04117958).

Strategies targeting DNA methylation and histone modification to treat GC majorly focus on inhibiting DNMTs and HDACs. Both DNMT inhibitors (such as 5-azacitidine and decitabine) and HDAC inhibitors (such as trichostatin A and valproic acid) can re-establish the expression of the tumor suppressor genes, particularly those involved in programmed cell death and therapeutic resistance. This gives them great potential for overcoming resistance by combination with chemotherapy and radiotherapy in GC treatments.^524^ In a phase I trial, the DNMT inhibitor 5-azacitidine was added to the neoadjuvant chemotherapy for GC. The treatment was well-tolerated with significant clinical and epigenetic responses.^629^ 5-azacitidine may be worth further investigation in more clinical trials. In a phase 2 trial, the HDAC inhibitor vorinostat was added to the standard capecitabine-cisplatin chemotherapy for first-line treatment of GC. The objective response rate was 42%, which is acceptable; however, more adverse events were observed in comparison with the historical data of fluoropyrimidine-platinium doublet regimens.^630^ Due to the lack of selectivity and the incomplete understanding of the pharmacology of these HDAC inhibitors, side effects are the main considerations. Comprehensive testing in preclinical models is needed before HDAC inhibitors can proceed to clinical trials.

**Fig. 5:**
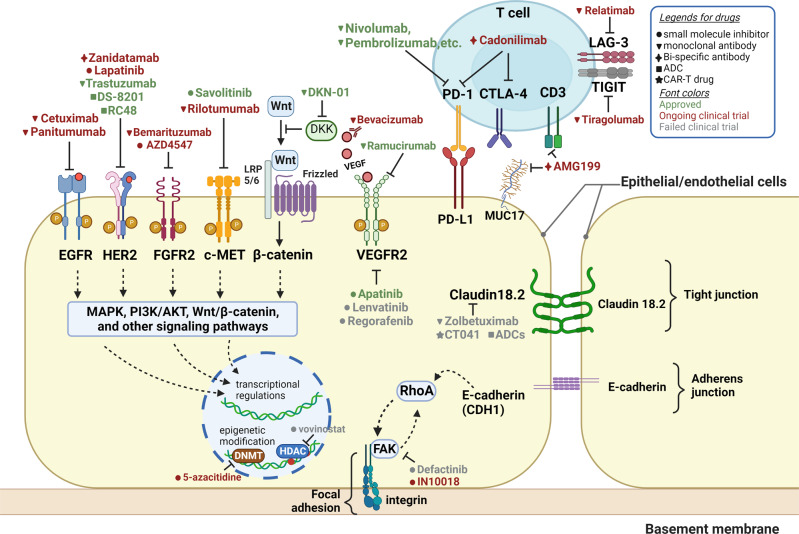
Overview of targeted therapy and immunotherapy in gastric cancer. The representative therapeutic targets in GC and the corresponding targeted or immunotherapeutic agents that have entered clinical investigations are depicted. EGFR epidermal growth factor receptor, MAPK mitogen-activated protein kinase, HER2 human epidermal growth factor receptor 2, PI3K phosphoinositide 3-kinases, FGFR2 fibroblast growth factor receptor 2, VEGFR2 vascular endothelial growth factor receptor 2, FAK focal adhesion kinase, RhoA Ras homolog family member A, PD-1 programmed death 1, PD-L1/2 programmed death ligand 1/2, ADC antibody-drug conjugate, LRP5/6 low-density lipoprotein receptor-related protein 5/6, DKK Dickkopf, CTLA-4 cytotoxic T-lymphocyte-associated protein 4, CD3 cluster of differentiation 3, TIGIT T cell immunoreceptor with Ig and ITIM domains, LAG-3 lymphocyte-activation gene 3, DNMT DNA methyltransferase, HDAC Histone deacetylases. This figure was created with Biorender.com

As summarized in Table 3 and Fig. 5, the development of growth factor or growth factor receptor antibodies, small molecule tyrosine kinase inhibitors, check point inhibitors, and adoptive immune cell therapies revolutionized treatment of GC. More novel therapies developed based on molecular biomarkers and signaling pathways are expected to improve precision medicine for GC.

**Table 3:** Collections of clinical trials related to targeted therapy and immunotherapy for gastric cancer

Study	Phase	Design	Patients	Target	Drug	Treatment	Number of patients	Line of therapy	Results
NCT01041404^40^	III	Randomized, open label, multi-center	Locally advanced, metastatic HER2 positive G/GEJ cancer	HER2	Trastuzumab	Arm1: Trastuzumab+chemotherapy; Arm2: chemotherapy	Arm1/Arm2: 294/290	1^st^	Improved OS
NCT03329690^582^	II	Randomized, open label, multi-center	Repeated-treated advanced HER2 positive G/GEJ cancer	HER2	Trastuzumab deruxtecan (DS-8201)	Arm1: DS-8201a; Arm2: irinotecan or paclitaxel	Arm1/Arm2: 125/62	After 2^nd^	Improved OS
NCT03556345^583,584^	II	Single arm, open label, single-center	Repeated-treated advanced HER2 positive GC	HER2	Disitamab vedotin (RC48)	RC48-ADC	125	2^nd^ or after 2^nd^	ORR is 24.8%, manageable safety
NCT02892123^585^	I	Non-randomized, open label, multi-center	Repeated-treated, locally advanced or metastatic HER2 positive cancers	HER2	Zanidatamab (ZW25)	Arm1: ZW25; Arm2: ZW25 + chemotherapy	Arm1/Arm2: 36/26	1^st^ or after 1^st^	ORR is 38% in Arm1 and 60% in Arm2
NCT00680901^587^	III	Randomized, quadruple blinded, multi-center	Locally advanced or metastatic HER2 positive G/GEJ cancer	HER2	Lapatinib	Arm1: CapeOx+lapatinib; Arm2: CapeOx+placebo	Arm1/Arm2: 249/238	1^st^	Failed to improve OS
NCT00678535^589^	III	Randomized, open label, multi-center	Locally advanced G/GEJ cancer	EGFR	Cetuximab	Arm1: cetuximab+capecitabine+cisplatin; Arm2: capecitabine+cisplatin	Arm1/Arm2: 455/449	1^st^	Failed to improve PFS
NCT00824785^590^	III	Randomized, open label, multi-center	Locally advanced or metastatic G/E/GEJ cancer	EGFR	Panitumumab	Arm1: EOX; Arm2: EOX + panitumumab	Arm1/Arm2: 275/278	1^st^	Failed to improve OS
NCT00548548^593^	III	Randomized, double blinded, multi-center	Locally advanced or metastatic GC	VEGF	Bevacizumab	Arm1: bevacizumab; Arm2: placebo	Arm1/Arm2: 387/387	1^st^	Failed to improve OS
NCT00917384^577^	III	Randomized, quadruple blinded, multi-center	Metastatic G/GEJ cancer	VEGFR2	Ramucirumab	Arm1: ramucirumab; Arm2: placebo	Arm1/Arm2: 238/117	2^nd^	Improved OS
NCT01512745^595^	III	Randomized, quadruple blinded, multi-center	Repeated-treated advanced or metastatic GC	VEGFR2	Apatinib	Arm1:apatinib; Arm2: placebo	Arm1/Arm2: 176/91	After 2^nd^	Improved OS
NCT01697072^599^	III	Randomized, triple blinded, multi-center	Untreated advanced MET positive G/GEJ cancer	c-MET	Rilotumumab	Arm1: rilotumumab; Arm2: placebo	Arm1/Arm2: 304/305	1^st^	Failed to improve OS
NCT02299648^601^	II	Single arm, open label, single-center	Metastatic or recurrent G/E/GEJ cancer	c-MET	Savolitinib	Savolitinib+docetaxel	25	2^nd^ or after 2^nd^	ORR is 28%
NCT01457846^602^	II	Randomized, open label, multi-center	Advanced G/GEJ cancer with FGFR2 polysomy or gene amplification	FGFR2b	AZD4547	Arm1: AZD4547; Arm2: paclitaxel	Arm1/Arm2: 40/27	2^nd^	Failed to improve PFS
NCT03343301,NCT03694522^603^	II	Randomized, double blinded, multi-center	Advanced G/GEJ cancer with FGFR2 overexpression or amplification	FGFR2b	Bemarituzumab (FPA144)	Arm1: bemarituzumab+mFOLFOX6; Arm2: placebo+mFOLFOX6	Arm1/Arm2: 77/78	1^st^	Improved PFS
NCT01630083^604^	II	Randomized, open label, multi-center	Advanced Claudin18.2 positive G/E/GEJ cancer	Claudin18.2	Zolbetuximab	Arm1: EOX; Arm2: EOX + zolbetuximab 800/600 mg/m^2 Arm3: EOX + zolbetuximab 1000 mg/m^2;	Arm1/Arm2/Arm3: 84/77/85	1^st^	Improved OS and PFS (Arm2 vs Arm1)
NCT03874897^605^	I	Single arm, open label, multi-center	Advanced Claudin18.2 positive G/GEJ and pancreatic cancer	Claudin18.2	CT041	CT041	37	2^nd^ or after 2^nd^	ORR is 48.6%, acceptable safety profile
NCT02267343^609^	III	Randomized, quadruple blinded, multi-center	Unresectable or recurrent G/GEJ cancer refractory to or intolerant of standard therapy	PD-1	Nivolumab	Arm1: nivolumab; Arm2: placebo	Arm1/Arm2: 330/163	After 2^nd^	Improved OS
NCT02872116^611^	III	Randomized, open label, multi-center	Untreated advanced HER2 negative G/E/GEJ cancer	PD-1	Nivolumab	Arm1: nivolumab+chemotherapy; Arm2: chemotherapy	Arm1/Arm2: 789/792	1^st^	Improved OS
NCT02494583^610^	III	Randomized, quadruple blinded, multi-center	Advanced G/GEJ cancer with PD-L1 CPS ≥ 1	PD-1	Pembrolizumab	Arm1: pembrolizumab; Arm2: pembrolizumab+chemotherapy; Arm3: placebo+chemotherapy;	Arm1/Arm2/Arm3: 256/257/250	1^st^	OS of pembrolizumab is non-inferior to chemotherapy
NCT03615326^612^	III	Randomized, quadruple blinded, multi-center	Untreated unresectable HER2 positive G/GEJ cancer	PD-1 and HER2	Pembrolizumab + trastuzumab	Arm1: pembrolizumab+trastuzumab+chemotherapy; Arm2: placebo+trastuzumab+chemotherapy	Arm1/Arm2: 217/217	1^st^	Improved ORR: Arm1: 74.4%, Arm2: 51.9%
NCT01585987^614^	II	Randomized, open label, multi-center	Unresectable or metastatic G/GEJ cancer	CTLA-4	Ipilimumab	Arm1: Ipilimumab; Arm2: best supportive care	Arm1/Arm2: 57/57	2^nd^	Failed to improve PFS
NCT03852251	Ib/II	Single arm, open label, single-center	Uptreated unresectable G/GEJ cancer	PD-1 and CTLA-4	Cadonilimab (AK104)	AK104 with or without chemotherapy	34	1^st^	ORR is 66.7% with acceptable safety profile
NCT05327231	Ib	Non-randomized, open label, multi-center	Previously treated locally advanced or metastatic G/GEJ cancer	FAK	IN10018	IN1001 with or without chemotherapy	33	2^nd^ or after 2^nd^	Ongoing
NCT04363801	IIa	Non-randomized, open label, multi-center	Advanced or metastatic G/GEJ cancer	DKK1	DKN-01	DKN-01+tislelizumab with or without chemotherapy	72	1^st^, 2^nd^	Ongoing
NCT04117958	I	Single arm, open label, multi-center	MUC17-positive solid tumors including G/GEJ	CD3 and MUC17	AMG 199	AMG 199	165	3^rd^	Ongoing
NCT01045538^630^	I/II	Single arm, open label, single-center	Unresectable GC	HDAC	Vorinostat	Vorinostat+capecitabine+cisplatin	45	1^st^	ORR is 42%, more adverse events
NCT01386346^629^	I	Single arm, open label, single-center	Advanced or metastatic gastric cancer	DNMT	Azacitidine	Azacitidine+chemotherapy	12	1^st^	ORR is 67%, well-tolerated

*HER2* human epidermal growth factor receptor 2, *OS* overall survival rate, *EGFR* epidermal growth factor receptor, *PFS* progression-free survival, *ORR* overall response rate, *VEGF* vascular endothelial growth factor, *VEGFR2* vascular endothelial growth factor receptor 2, *c-MET* tyrosine-protein kinase mesenchymal-epithelial transition factor, *FGFR2b* fibroblast growth factor receptor 2b, *PD-1* programmed death-ligand 1, *CTLA-4* cytotoxic T-lymphocyte-associated protein 4, *GEJ* gastroesophageal junction, *IHC* intrahepatic cholestasis, *ADC* antibody-drug conjugate, *CapeOx/XELOX* oxaliplatin + capecitabine, *EOX* epirubicin + oxaliplatin + capecitabine, *FOLFOX* leucovorin + 5-FU + oxaliplatin, *CAR* chimeric antigen receptor, *FAK* focal adhesion kinase, *DKK* Dickkopf, *CD3* cluster of differentiation 3, *HDAC* histone deacetylases, *DNMT* DNA methyltransferase.

## Summary and perspectives

Compared to chemotherapy, targeted therapy for GC is safer and more effective. Some molecular-targeted drugs such as trastuzumab and apatinib have also been approved for the treatment of GC. The development of more effective drugs and the search for biomarkers with stronger sensitivity and specificity are still major challenges in the targeted treatment of GC. Owing to the interpatient and intratumor heterogeneity of GC, developing personalized therapy for GC patients has been the main demand in contemporary combat against GC. With the advent of technologies for genome-wide analysis and the establishment of novel preclinical models, treatment of GC has been moving toward precision medicine. The molecular classifications of GC enable more personalized targeted therapies and immunotherapies for GC patients and greater understanding of the molecular mechanisms underlying GC development, progression, metastasis, and therapeutic resistance. This has shed light on novel diagnosis/prognosis biomarkers and potential therapeutic targets. Principal signaling pathways mentioned here include MAPK, HER2, PI3K/AKT/mTOR, p53, Wnt/β-catenin, NF-κB, TGF-β, HGF/c-MET signaling pathways, and those involved in immunomodulation. Other signaling pathways with relatively limited research, such as FGF-FGFR, STAT3, HIF-1α, Hedgehog, and Notch signaling pathways, and the cell adhesion/junction-related signaling molecules, have also been discussed for molecular mechanisms and potential therapeutic targets. Among the identified targets from the molecular discoveries, several have at least entered phase II clinical investigations. These include HER2, EGFR, VEGFR, FGFR2, Claudin18.2, Trop2, c-MET, and the immune checkpoint molecule PD-1. However, the molecular mechanisms are generally not associated with a unique signaling pathway but with crosstalk or feedback loops. Bypass pathways are critical contributors to therapeutic resistance when mono-targeted therapy is used. Therefore, the development and verification of novel combination regimens are in urgent demand.

The immune checkpoint inhibitor PD-1 monoclonal antibody has been approved for the first-line treatment of GC. Recently, 18 patients with rectal cancer received nine doses of dostarlimab (a PD-1 blocker) intravenously for immunotherapy. After 6 months of treatment, all 18 patients achieved complete clinical remissions.^631^ This study strongly demonstrates that immunotherapy is the future trend to treat gastrointestinal tumors. Immunotherapy has good safety and a durable immune response. With the rapid development of the high-throughput and whole-exome sequencing for immunologic screening of mutant genes, more neoantigen-reactive tumor-infiltrating lymphocytes (TIL) will be identified in GCs, which means more specific immunogenic gene products can be developed. Therefore, traditional therapy combined with immunotherapy is the trend in GC treatment. The timing of immunotherapy, the selection of drug combinations and combined therapy dose, the management of treating-related adverse events, and the selection of biomarkers for predicting clinical efficacy all need further research, but it shows a good prospect in the treatment of GC.

**Fig. 6:**
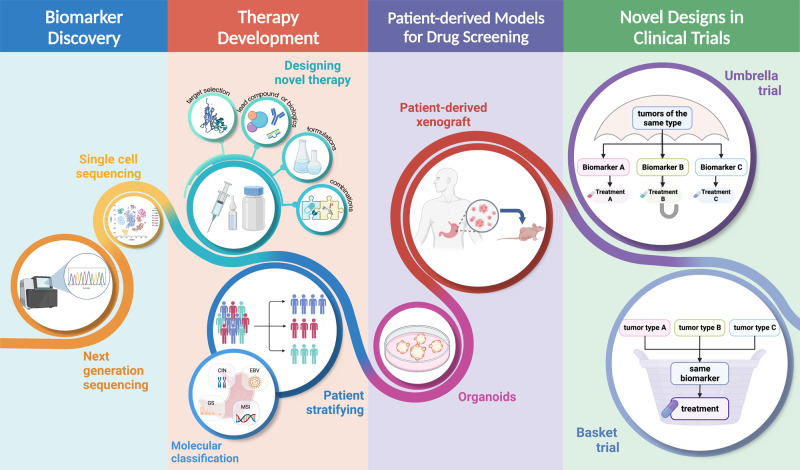
Essential technologies and processes for elevating biomarker-guided precision medicine. The next-generation sequencing and novel technologies like single cell sequencing for profiling genetic changes enable biomarker identification with higher precision. Biomarkers are the basis for molecular classification and patient stratifying. Meanwhile, biomarker-based novel therapy is developed as the target is selected. New therapeutic agents are developed with lead compound or biologics identified, followed by formulation optimization and possible combination designs. The patient-derived xenograft or organoid research models are useful tools for drug screening and molecular mechanism verifications. Finally, novel clinical trial designs like umbrella trials and basket trials enable precise evaluation of treatment effects under a fine stratification of patients. This figure was drafted with Biorender.com and modified using Adobe Photoshop

Although the systematic treatment of GC has evolved rapidly in recent years, there are still limited drugs available in the clinic. Innovation is needed to speed up drug development for GC. We expect breakthroughs to be made in GC therapy by looking deep into the tumor microenvironment specific to GC, stratifying patients more precisely using next-generation sequencing (NGS), and individualizing treatment through organoid-based functional drug predictions. NGS, like whole-exome sequencing (WES), and novel technologies, like single cell sequencing for profiling genetic changes, are the basis for biomarker identification and precision medicine. However, the complexity of NGS data analysis and its high cost hinder its application in the clinic. It is important to lower the cost of clinical NGS sequencing and expand its use to cover most of the GC patients. This will help the discovery of low-frequency genetic aberrations and the development of novel therapies. The complexity of cancer genomics requires fine stratification of patients to receive corresponding drugs. This means there are few patients to receive each drug treatment, which hinders the evaluation of the treatment effect. The umbrella trial and the basket trial were designed to deal with this issue.^632^ In an umbrella trial, patients with the same type of cancer are stratified into different subgroups based on their molecular profiles, and patients in each subgroup are treated accordingly. In a basket trial, patients with different types of tumors but the same targets are grouped. The drug of interest is tested in this phenotype-heterogeneous but genotype-homogeneous group of patients. These two novel designs for clinical trials have been used for new drug discovery and personalized cancer treatment (Fig. 6). For example, the VIKTORY trial was the largest umbrella trial in GC, where GC patients were assigned to eight different biomarker groups based on NGS.^601^ This study demonstrated the efficacy of targeted therapy in certain molecular subgroups.

However, biomarker-based precision medicine is restricted by the low sensitivity and specificity of biomarkers in predicting sensitivity or resistance to drugs. In addition, most of the patients lack actionable targets even after extensive biomarker profiling. As an alternative to biomarker-based drug prediction, functional drug screening may be a new strategy for cancer precision medicine. The organoid technology combines stem cell niche and 3D extracellular matrix (ECM) for in-vitro cell culture. Stem cells can form organized cell structures resembling their tissue-of-origin at both cellular and structural levels. When cancer cells derived from human tumors are cultured under organoid conditions, they can be expanded and stably passaged like cancer cell lines. The tumor organoid can faithfully maintain the genotypes and phenotypes of the original tumor tissue. Most importantly, the tumor organoid also maintains the sensitivity of the original tumor to drugs. These features make the organoid an ideal tool for in-vitro functional drug screening. Observational studies have confirmed the consistency of organoid-based drug sensitivity to the clinic response of the patients receiving the same regime.^633–635^ Researchers around the world, including us, have been trying to establish patient-derived organoids to guide GC treatment.^636–638^ It is hoped that organoid-based drug screening will go from bench to bedside to benefit cancer patients.

Recognition of novel molecular targets has also paved the way for developing gene therapy as a promising molecular alternative in GC treatment, including gene silencing approaches to inactivate oncogenes, replacing defective tumor suppressor genes, introducing suicide genes, genetic immunotherapy, and so forth. The therapeutic potential of genetic approaches has been demonstrated in certain in vitro studies, such as a nanoparticle-delivered siRNA to suppress oncogene CFL1^639^ and a CRISPR/Cas9 system-delivered LncRNA PANDAR (promoter of CDKN1A antisense DNA damage activated RNA) to interact with p53 and competitively regulate *CDKN1A* transcription in GC cell lines.^640^ Like drug-based therapies, the major challenge of gene therapy lies in finding a way to circumvent non-responsiveness, which is caused by immunogenic effects after the delivery of genetic material. A newly published study reported that combining p53 mRNA nanotherapy with anti-PD-L1 therapy can reprogram the immune microenvironment for improved anti-cancer effects compared to monotherapy.^641^ This implies that proper formulation and combination design with an optimized delivery system will be the key to developing novel targeted therapy, immunotherapy as well as gene therapy that can circumvent therapeutic tolerance or resistance.

Beyond any doubt, early diagnosis and effective prevention strategies are indispensable to reducing the morbidity and mortality of GC. Lifestyle control and endoscopic screening have been useful prevention approaches. As *H. pylori* infection is the dominant risk factor for GC development, testing for *H. pylori* and chemo-eradication have been the primary prevention strategy for GC.^642^ Additionally, vaccines aimed at eradicating *H. pylori* are under development.^643^ For early medication managements, the identification of novel molecular markers driven by the NGS technologies could improve precision in both diagnosis and therapeutic interventions.
